# Can corrections spread misinformation to new audiences? Testing for the elusive familiarity backfire effect

**DOI:** 10.1186/s41235-020-00241-6

**Published:** 2020-08-26

**Authors:** Ullrich K. H. Ecker, Stephan Lewandowsky, Matthew Chadwick

**Affiliations:** 1 School of Psychological Science (M304), University of Western Australia, 35 Stirling Hwy, Perth, WA 6009 Australia; 2 School of Psychological Science, University of Bristol, 12a Priory Road, Bristol, BS8 1TU UK

**Keywords:** Continued influence effect, Fact-checking, Myth debunking, Familiarity backfire effect, Illusory truth effect, Mere exposure effect

## Abstract

Misinformation often continues to influence inferential reasoning after clear and credible corrections are provided; this effect is known as the continued influence effect. It has been theorized that this effect is partly driven by misinformation familiarity. Some researchers have even argued that a correction should avoid repeating the misinformation, as the correction itself could serve to inadvertently enhance misinformation familiarity and may thus backfire, ironically strengthening the very misconception that it aims to correct. While previous research has found little evidence of such familiarity backfire effects, there remains one situation where they may yet arise: when correcting entirely novel misinformation, where corrections could serve to spread misinformation to new audiences who had never heard of it before. This article presents three experiments (total *N* = 1718) investigating the possibility of familiarity backfire within the context of correcting novel misinformation claims and after a 1-week study-test delay. While there was variation across experiments, overall there was substantial evidence against familiarity backfire. Corrections that exposed participants to novel misinformation did not lead to stronger misconceptions compared to a control group never exposed to the false claims or corrections. This suggests that it is safe to repeat misinformation when correcting it, even when the audience might be unfamiliar with the misinformation.

## Significance statement

Misinformation often continues to influence people’s thinking and decision-making even after they have received clear, credible corrections; this is known as the continued influence effect. It has been suggested that this effect is partly driven by the familiarity of false claims, such that people are particularly influenced by false claims that seem especially familiar (“I have heard that before, so there must be something to it!”). Some researchers have even recommended that a correction should avoid repeating the misinformation, out of concerns that the correction itself could inadvertently enhance the familiarity of the false claim. This could lead to corrections backfiring, ironically strengthening the very misconceptions that they aim to correct. While previous research has found little evidence of such familiarity backfire effects, there remains one situation where they may yet arise: when correcting entirely novel misinformation. Such corrections might familiarize people with false claims that they had never encountered before, and, therefore, such corrections could serve to spread misinformation to new audiences. This article presents three online experiments (total *N* = 1718 participants) investigating the possibility of familiarity backfire within the context of correcting novel misinformation claims. While there was some variation across experiments, overall there was substantial evidence *against* familiarity backfire: Corrections that exposed participants to novel misinformation did not lead to stronger misconceptions compared to a control group never exposed to the false claims or corrections. This suggests that it is safe to repeat misinformation when correcting it, even when the audience might be unfamiliar with the misinformation.

The advent of the Internet and the subsequent rise of social media as a primary form of communication has facilitated the distribution of misinformation at unprecedented levels (Southwell & Thorson, 2015; Vargo, Guo, & Amazeen, 2018). Misinformation can have detrimental effects at a societal and individual level, as ill-informed decisions can have negative economic, social, and health-related consequences (Bode & Vraga, 2018; Lazer et al., 2018; Lewandowsky, Ecker, & Cook, 2017; MacFarlane, Hurlstone, & Ecker, 2020; Southwell & Thorson, 2015). This is concerning because there is a significant disparity between the ease of disseminating misinformation and the difficulty of correcting it. Corrections can be ineffective, and individuals often continue to use corrected misinformation in their inferential reasoning, a phenomenon termed the continued influence effect (Chan, Jones, Hall Jamieson, & Albarracín, 2017; Johnson & Seifert, 1994; Lewandowsky, Ecker, Seifert, Schwarz, & Cook, 2012; Paynter et al., 2019; Rich & Zaragoza, 2016; Walter & Tukachinsky, 2020; Wilkes & Leatherbarrow, 1988).

One theoretical account of the continued influence effect assumes that it results from selective retrieval (Ecker, Lewandowsky, Swire, & Chang, 2011; Ecker, Lewandowsky, & Tang, 2010; Gordon, Quadflieg, Brooks, Ecker, & Lewandowsky, 2019; Swire, Ecker, & Lewandowsky, 2017). More specifically, in line with dual-processing models of memory (e.g., Diana, Reder, Arndt, & Park, 2006; Yonelinas & Jacoby 2002; Zimmer & Ecker, 2010), continued influence effects might arise when a reasoning task features a retrieval cue that automatically activates the misinformation, while recollection of the correction fails (see Ayers & Reder, 1998; Marsh & Fazio, 2006). According to this account, automatic misinformation activation is driven by familiarity, an automatic process that facilitates the rapid, context-free retrieval of previously encountered stimuli, whereby the degree of activation of a memory representation depends upon the frequency with which the associated stimulus has been encountered in the past (Hintzman & Curran, 1994).

It follows that one driver of the continued influence effect may lie in the fact that misinformation is typically repeated within a correction, boosting its familiarity—for example, clarifying that vaccines do *not* cause autism all but requires repetition of the false vaccine-autism association (e.g., see Nyhan, Reifler, Richey, & Freed, 2014; Paynter et al., 2019). Apart from the fact that enhanced familiarity will facilitate automatic misinformation retrieval, familiarity has also been found to foster perceived truthfulness via metacognitive processes (Begg, Anas, & Farinacci, 1992; Dechêne, Stahl, Hansen, & Wänke, 2010; Parks & Toth, 2006)—either because enhanced familiarity indicates greater social consensus (Weaver, Garcia, Schwarz, & Miller, 2007; also see Arkes, Boehm, & Xu, 1991) or because familiar information is processed more fluently and the perceived fluency is misattributed to the information’s validity (Pennycook, Cannon, & Rand, 2018; Schwarz, Sanna, Skurnik, & Yoon, 2007; Unkelbach, 2007). Thus, corrections that repeat the misinformation might inadvertently increase the likelihood of it being retrieved and perceived as valid in subsequent reasoning tasks (Schwarz et al., 2007; Swire et al., 2017).

It has even been suggested that the boost in familiarity associated with the repetition of misinformation within a correction could be so detrimental that it could ironically *increase* belief in the corrected misinformation (Schwarz et al., 2007). This increase in post-correction belief in misinformation, relative to either a pre-correction baseline in the same sample of participants, or a no-misinformation-exposure baseline in a separate sample, has been termed the familiarity backfire effect (Cook & Lewandowsky, 2011; Lewandowsky et al., 2012). In order to avoid this effect, it is commonly suggested to educators, journalists, and science communicators that corrections should avoid repeating the targeted misinformation as much as possible (Cook & Lewandowsky, 2011; Lewandowsky et al., 2012; Peter & Koch, 2016; Schwarz, Newman, & Leach, 2016; Schwarz et al., 2007).

However, despite familiarity backfire effects being prominently discussed in the literature, empirical evidence of such effects is scarce. In fact, the only clear demonstration of a familiarity backfire effect was reported in an unpublished manuscript by Skurnik, Yoon, and Schwarz (2007; discussed by Schwarz et al., 2007), who presented participants with a flyer juxtaposing “myths vs. facts” associated with the flu vaccine. It was found that after a 30-min delay, a substantial proportion of myths were misremembered as facts, and that attitudes towards the flu vaccine became more negative compared to participants who had not been presented with the flyer. In a similar study, Skurnik, Yoon, Park, and Schwarz (2005) found that participants were more likely to misremember myths as facts after repeated vs. singular retractions. However, these effects were only found with a 3-day test delay and only in older adults (not after shorter delays and in younger adults, as in Skurnik et al., 2007), and the study also did not feature a baseline condition against which to access actual “backfire.”

By contrast, a number of contemporary studies have failed to find evidence of familiarity backfire effects. For example, unlike Skurnik et al. (2005), Ecker et al. (2011) found that multiple retractions were more effective than singular retractions at reducing continued influence. Cameron et al. (2013) compared the effectiveness of flu-vaccine myth corrections that either avoided misinformation repetition (presenting facts only) or repeated misinformation (including one condition featuring Skurnik et al.’s (2007) “myths vs. facts” flyer). Flu-vaccine knowledge was measured prior to the manipulation and again after a week, together with post-intervention belief in the true and false claims. Cameron et al. found that all conditions were successful at reducing misconceptions, with the best outcomes in the “myths vs. facts” condition, and the worst outcomes in the facts-only condition that avoided myth repetition. Likewise, Ecker, Hogan, and Lewandowsky (2017) found that repeating a piece of misinformation when correcting it actually led to stronger reduction of the continued influence effect than a correction that avoided misinformation repetition. They argued that misinformation repetition fosters co-activation of the misinformation and its correction, which in turn facilitates conflict detection and information integration when the correction is encoded, leading to stronger knowledge revision (see Kendeou, Walsh, Smith, & O’Brien, 2014). Finally, Swire et al. (2017) presented participants with a series of true and false claims that were subsequently affirmed or corrected and measured the corresponding change in belief. They, too, failed to observe any familiarity backfire effects: post-correction belief in misinformation was always lower than pre-correction belief. This reduction in false-claim belief was observed even under conditions where the impact of familiarity (relative to recollection) should be maximal, viz. in elderly participants and after a long retention interval of up to 3 weeks. Swire et al. concluded that familiarity may contribute to continued influence effects (i.e., ongoing reliance on corrected misinformation, especially after a delay, when recollection of the correction fades but familiarity of the misinformation remains relatively intact; see Knowlton & Squire, 1995), but that misinformation familiarity is not typically associated with backfire effects (i.e., ironic boosts to false-claim beliefs relative to a pre-correction or no-exposure baseline).

In a recent study, Ecker, O’Reilly, Reid, and Chang (2020) found that presenting participants with only a correction (a brief retraction or a more detailed refutation) of a real-world false claim, without prior exposure to the false claim itself, decreased both false-claim-congruent reasoning and belief in the false claim relative to a control group who received no exposure to the claim. This demonstrated that mere exposure to a false claim within a correction did not cause a familiarity backfire effect. However, Ecker et al. highlighted one remaining situation where a familiarity backfire effect may yet occur: when *novel* misinformation is introduced to a recipient through a correction. If a person’s first encounter with a false claim is provided by a correction, the correction could inadvertently familiarize the person with the previously unfamiliar misinformation; corrections may thus potentially spread the misinformation to new audiences (as suggested by Schwarz et al., 2016). Indeed, the greatest boost to a claim’s familiarity will be associated with the initial encounter, while additional encounters will bring about exponentially decreasing familiarity boosts (consistent with theoretical frameworks that propose novelty-dependent encoding; e.g., Oberauer, Lewandowsky, Farrell, Jarrold, & Greaves, 2012).

It is easy to see how social media could facilitate situations where an individual is exposed to a correction without previously having encountered the corresponding misinformation. Such exposure may not only familiarize the consumer with the novel misinformation, but may also lend some credibility to the false claim, in the sense that a correction may signal that someone actually believes the false claim to be true, thus warranting a correction. This makes the possibility of a familiarity backfire effect with novel misinformation a concerning notion. Thus, the main purpose of the present study was to investigate the possibility of a familiarity backfire effect within the context of correcting novel misinformation. To this end, the study aimed to replicate Ecker et al. (2020), using claims that were maximally novel to participants.

Except for the use of novel false claims, Experiment 1 was a straight replication of the brief-retraction conditions of Ecker et al. (2020; Experiment 2). Experiments 2 and 3 aimed to replicate Experiment 1, while manipulating factors that should influence the relative impact of familiarity, viz. retention interval (Experiment 2) and cognitive load during encoding (Experiment 3).

## Experiment 1

**Fig. 1:**
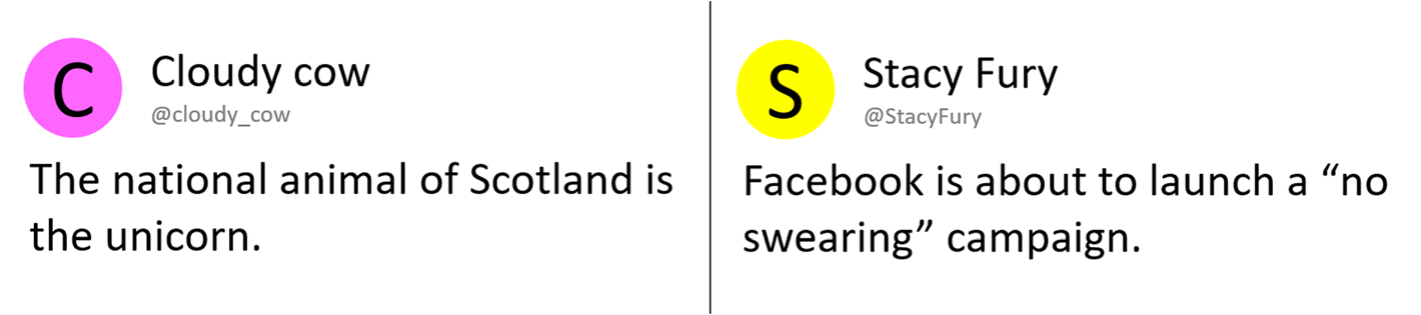
Example of a true claim (left) and false claim (right)

**Fig. 2:**
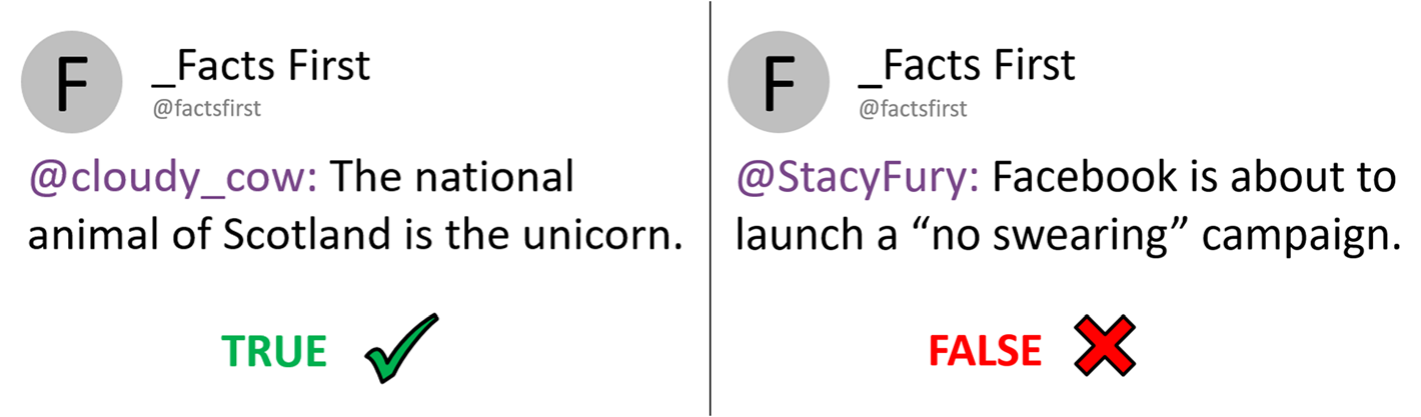
Example of an affirmation (left) and correction (right)

Experiment 1 presented participants with true and false claims and/or associated affirmative or corrective fact-checks. An example claim was “The national animal of Scotland is the unicorn” (see Fig. 1 and the “Method” section for further details). The experiment used a 2 × 2 between-subjects design, fully crossing factors claim exposure (yes/no) and fact-check exposure (yes/no).^1^ Conditions were no-exposure control (NE), claim-only (CO), fact-check-only (FCO), and claim-plus-fact-check (CFC; in this condition, participants first received all claims without any indication of validity, and then received the fact-checks separately). The experiment was designed to encourage participants to rely on familiarity during retrieval in order to maximize the possibility of observing familiarity-related backfire effects. Fact-checks in fact-check-only and claim-plus-fact-check conditions therefore simply stated the claim with a brief affirmation or correction (e.g., “The national animal of Scotland is the unicorn” followed by the word “TRUE” and a green tick mark; see Fig. 2) but did not provide supporting, detailed information, since additional refutational information has been shown to increase the likelihood that the corrective message is later recollected (Chan et al., 2017; Ecker et al., 2020; Paynter et al., 2019; Swire et al., 2017). Additionally, a 1-week retention interval between exposure and test was used, as the ability to engage in recollection diminishes over time, while familiarity remains relatively constant (Knowlton & Squire, 1995).

Belief in the claims at test was determined by direct claim-belief ratings, as well as a series of inference questions that indirectly measured claim belief by assessing claim-congruent reasoning. The inference questions were presented first because the inference score was determined a priori as the main dependent variable of interest, following ample precedent (e.g., Ecker et al., 2017). The inference score provides a belief measure that is not “contaminated” by concurrent exposure to the core claim, whereas it is impossible to measure direct belief in a claim without at the same time exposing participants to it. Thus, only the inference score provides a “clean” baseline in the no-exposure condition. Moreover, presenting the claims for a direct belief rating first would have artificially increased claim familiarity across all conditions, and acted as a potent retrieval cue for recollection of the fact-checks. The core hypothesis (H1_FIS_)^2^ was that we would observe a familiarity backfire effect; that is, that mere exposure to corrective fact-checks would lead to increased inference scores relative to the no-exposure baseline (i.e., NE < FCO).

A series of secondary hypotheses was specified as follows (these are also summarized, together with the primary hypothesis, in Table 1 in the “Results” section):

Hypothesis H1_FBR_ was that mere corrections would also increase false-claim belief ratings relative to baseline (i.e., NE < FCO). Hypothesis H1_TIS/TBR_ was that mere affirmations would be effective and would thus increase inference scores and true-claim belief ratings relative to baseline (i.e., NE < FCO).

Hypothesis 2 investigated the illusory truth effect, whereby mere exposure to information renders it more likely to be evaluated as truthful (Dechêne et al., 2010). It was specified that mere exposure to claims would increase claim-congruent reasoning for both false claims (H2_FIS_) and true claims (H2_TIS_), and boost belief in both false (H2_FBR_) and true claims (H2_TBR_), relative to baseline (i.e., NE < CO).

Hypothesis 3 tested the effectiveness of fact-checking a claim that had already been encountered. It was specified that, relative to the claims-only condition, fact-checks of previously presented claims would decrease false-claim-congruent reasoning and false-claim belief (i.e., CFC < CO; H3_FIS_ and H3_FBR_), while increasing true-claim-congruent reasoning and true-claim belief (i.e., CFC > CO; H3_TIS_ and H3_TBR_).

Finally, Hypothesis 4 tested whether correcting previously presented false claims would reduce inference and belief scores back to or even below baseline. This is technically a test for continued influence, as previous research has found that corrections are often not able to eliminate misinformation influence down to baseline levels. However, in most continued-influence studies, the misinformation is initially presented as true and valid, whereas the initial presentation of false claims in the claim-plus-fact-check condition occurred without validation (i.e., the false claim was presented initially without being labeled a fact, which would have presumably increased initial belief, making it harder to subsequently bring belief back down to baseline). It was, therefore, not expected that inference scores would be greater in the claim-plus-fact-check condition than the no-exposure control. In fact, guided by the results of Ecker et al. (2020), we expected that corrections of previously presented false claims would decrease false-claim-congruent reasoning (H4_FIS_) and false-claim belief (H4_FBR_) back to or even below the level of the no-exposure control, and specified Hypothesis 4 as NE > CFC.

### Method

#### Participants

An a-priori power analysis using G*Power3 (Faul, Erdfelder, Lang, & Buchner, 2007) indicated that a minimum sample size of 352 was needed to detect a small effect of *f* = .15 between two groups with *α* = .05 and 1 − *β* = .80. In order to account for attrition rates and ensure sufficient power, it was decided to recruit 440 participants—however, due to miscommunication, this sample size was used for the entire experiment even though its calculation was based on only two groups, and thus the experiment was somewhat underpowered. Participants were US-based adult Amazon Mechanical Turk (MTurk) workers, who had completed at least 5000 so-called human-intelligence tasks (HITs) with 97% + approval. MTurk data are largely regarded as being of comparative quality to data from convenience samples (Berinsky, Huber, & Lenz, 2012; Hauser & Schwarz, 2016; Necka, Cacioppo, Norman, & Cacioppo, 2016).

A subset of 331 participants was randomly assigned to one of the three exposure conditions (CO, FCO, or CFC) of an experimental survey, with the constraint of approximately equal cell sizes. The retention rate between study and test was approximately 80%, with 264 participants returning for the test phase. An additional 109 participants completed the NE control condition, which involved only a test phase and was, therefore, run separately (and concurrently with the test phase of the other conditions). Two participants were identified as erratic responders based on an a-priori exclusion criterion (see “Results” for details). The final sample size for analysis was thus *N* = 371 (condition NE *n* = 108; CO *n* = 81; FCO *n* = 92; CFC *n* = 90; age range 20–71 years; *M*_age_ *=* 39.91; *SD*_age_ = 11.99; 208 male, 160 female, and 3 participants of undisclosed gender). A post-hoc power analysis confirmed an achieved power in regards to the observed main effect of condition in the analysis of inference scores (*η*_*p*_^2^ = .022; see “Results” below) of 1 − *β* = .67. Participants were paid US$0.40 for the study phase and US$0.60 for the test phase.

#### Materials

##### Claims

A total of 12 claims (six true, six false) were selected from an initial pool of 48, with the intention of minimizing claim familiarity. To this end, prior to conducting the present study, the 48 claims were evaluated by a separate sample of *N* = 91 participants via an MTurk survey (see “Appendix” for details). The familiarity and believability of each claim were rated on Likert scales ranging from 1 (low familiarity/believability) to 5 (high familiarity/believability). Claims with familiarity ratings > 2 were excluded, as were excessively believable or unbelievable claims (believability ratings < 2 or > 4), resulting in a pool of 22 candidate claims. From this pool, the least familiar claims were then selected while taking into account additional factors such as comprehensibility and the quality of corresponding inferential-reasoning questions that could be generated. All claims are provided in the “Appendix.” The average familiarity of selected false claims was *M* = 1.67, with mean believability of *M* = 2.89; average familiarity of selected true claims was *M* = 1.63, with mean believability of *M* = 2.54.

Claims were presented in a format that mimicked a social-media post (see Fig. 1). Each claim was associated with a different fictional account, and was displayed underneath the account name. A circular image with the first letter of the account handle was displayed instead of a traditional profile picture, similar to the default icon for a Google account.

##### Fact-checks

There were 12 fact-checks matched to the 12 claims; these were displayed in the same social-media format as the original claim (see Fig. 2). Each fact-check repeated the corresponding claim along with an affirmation (a “TRUE” tag and a green tick) if the claim was true, or a correction (a “FALSE” tag and a red cross) if it was false. All fact-checks were associated with the fictional account “Facts First,” which was introduced as an independent and objective fact-checking group that verifies claims on social media.

##### Measures

Claim-related inferential reasoning was measured through a series of 24 inference questions designed to indirectly assess claim beliefs. There were two such questions per claim, one of which was reverse-coded. Each item presented the participants with a statement that was related to a claim, but did not repeat the claim itself. Statements were designed such that agreeing or disagreeing with them would require reasoning that is congruent or incongruent with belief in the original claim. An example item was “Facebook is investing money into promoting inoffensive language on its platform.” Participants were asked to rate their level of agreement with each statement on a Likert scale ranging from 0 (complete disagreement) to 10 (complete agreement). Inference questions are provided in the “Appendix.” Claim belief was additionally measured through 12 direct belief ratings. Participants were asked to indicate how much they believed each claim to be true or false on a Likert scale ranging from 0 (certainly false) to 10 (certainly true).

#### Procedure

The experiment was administered using Qualtrics survey software (Qualtrics, Provo, UT) via the CloudResearch platform (formerly TurkPrime; Litman, Robinson, & Abberbock, 2017). After being presented with an ethically approved information sheet, participants answered demographic questions regarding their English language proficiency, gender, age, and country of residence. In the study phase, depending on experimental condition, participants read either a series of claims (claim-only condition CO), a series of fact-checks (fact-check only, FCO), or a series of claims followed by a series of associated fact-checks (claim-plus-fact-check, CFC). All claims and/or fact-checks were presented individually for at least 3 s. After a 1-week retention interval, participants who completed the study phase were invited by email to participate in the test phase. Participants in the no-exposure condition (NE) only completed the test phase. In the test phase, participants were first presented with the 24 inference questions. Inference questions were grouped by claim (i.e., paired questions were always presented together), but otherwise the sequence was randomized. Participants then answered the 12 direct belief questions in a random order. Finally, participants were asked if they had put in a reasonable effort and whether their data should be used for analysis (with response options “Yes, I put in reasonable effort”; “Maybe, I was a little distracted”; or “No, I really wasn’t paying any attention”), before being debriefed.

### Results

Data from all experiments are available at https://osf.io/69bq3/. Before analysis, we applied a set of a-priori exclusion criteria. Three criteria were not met by any participants, namely English proficiency self-rated as “poor”, uniform responding (identified by a mean *SD* <  0.5 across all responses), and self-reported lack of effort (“no” response to the effort question). To identify erratic responding, we applied the following procedure: After inverting all reverse-keyed items such that greater inference scores reflected stronger claim-congruent reasoning, for each claim we calculated the mean absolute difference between the two inference-question responses (IQ1 and IQ2) and the belief rating (BR) as (|*IQ1* – *IQ2|* + |*IQ1* – *BR*| + |*IQ2* – *BR*|)/3. The mean absolute differences across all 12 claims were then averaged to produce a final score, where entirely consistent responding would result in values approaching zero. This score was then used to identify and reject erratic responders, using the inter-quartile outlier rule with a 2.2 multiplier (Hoaglin & Iglewicz, 1987). As mentioned earlier, we excluded *n* = 2 erratic responders based on this procedure.

Mean false-claim and true-claim inference scores were calculated by averaging the scores associated with the 12 false-claim and 12 true-claim inference questions, respectively. Inference scores ranged from 0 to 10. The belief ratings associated with the six false claims were averaged to create a false-claim belief rating, and the ratings associated with the six true claims were averaged to create a true-claim belief rating. The scale was 0–10.

#### False claims

##### False-claim inference scores

**Fig. 3:**
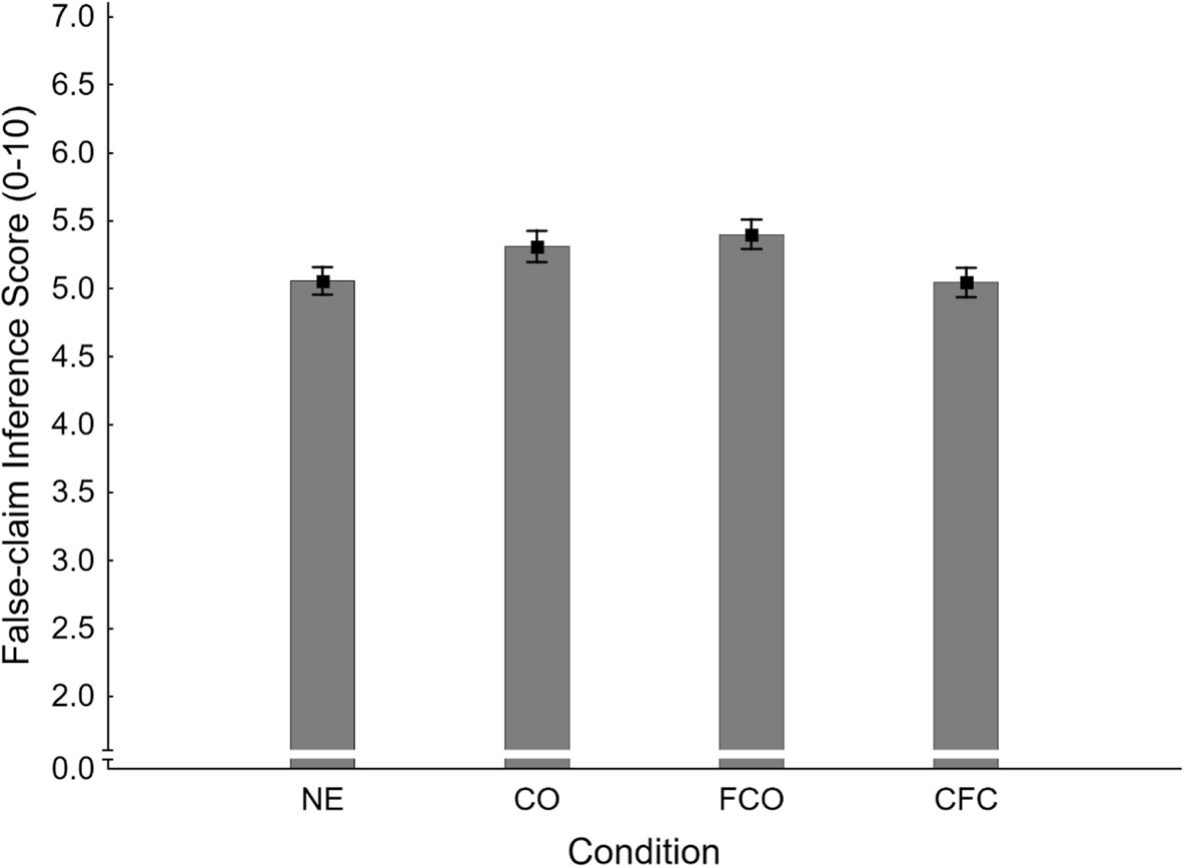
Mean false-claim inference scores across conditions NE (no-exposure), CO (claim-only), FCO (fact-check-only), and CFC (claim-plus-fact-check) in Experiment 1. Error bars show standard errors of the mean

Mean false-claim inference scores across conditions are shown in Fig. 3. A one-way analysis of variance (ANOVA) revealed a small but significant main effect of condition, *F*(3,367) = 2.73, *η*_*p*_^2^= .022, *p* = .044. To test the primary hypothesis that corrections of novel myths would produce a familiarity backfire effect, a planned contrast compared no-exposure (NE *M* = 5.06, *SE* = 0.10) and fact-check-only conditions (FCO *M* = 5.40, *SE* = 0.11). This contrast was significant, *F*(1,367) = 5.31, *η*_*p*_^2^ = .014, *p* = .022. Thus, a small familiarity backfire effect was observed, and H1_FIS_ was supported.

Next, three secondary planned contrasts were conducted on false-claim inference scores, applying the Holm-Bonferroni correction (Holm, 1979). The results of these contrasts are reported in the first panel of Table 1 (together with the primary contrast). In order to test for an illusory truth effect, we compared the claim-only (CO *M* = 5.31, *SE* = 0.12) and no-exposure conditions. The difference was non-significant, and H2_FIS_ was rejected accordingly.

**Table 1:** Contrasts run in Experiment 1

dV/hypothesis	Effect tested	*F*(1,367)	*P*
False-claim inference scores			
**H1**_**FIS**_**: NE < FCO**	**Familiarity backfire effect**	**5.31**	**.022**^**a**^
H2_FIS_: NE < CO	Illusory truth effect	2.72	.100
H3_FIS_: CFC < CO	Effect of claim+correction vs. claim-only	2.73	.099
H4_FIS_: NE > CFC	Effect of claim+correction vs. baseline	0.01	.941
False-claim belief ratings			
H1_FBR_: NE < FCO	Familiarity backfire effect	< 0.01	.971
H2_FBR_: NE < CO	Illusory truth effect	3.03	.082
H3_FBR_: CFC < CO	Effect of claim+correction vs. claim-only	13.75	<.001^a^
H4_FBR_: NE > CFC	Effect of claim+correction vs. baseline	4.78	.029
True-claim inference scores			
H1_TIS_: NE < FCO	Effect of affirmation vs. baseline	36.09	<.001^a^
H2_TIS_: NE < CO	Illusory truth effect	4.23	.041^a^
H3_TIS_: CFC > CO	Effect of claim+affirmation vs. claim-only	9.40	.002^a^
True-claim belief ratings			
H1_TBR_: NE < FCO	Effect of affirmation vs. baseline	82.84	<.001^a^
H2_TBR_: NE < CO	Illusory truth effect	5.32	.022^a^
H3_TBR_: CFC > CO	Effect of claim+affirmation vs. claim-only	30.95	<.001^a^

*Note.* Hypotheses are numbered H1–4 (primary hypothesis in bold; see text for details); subscripts FIS, TIS, FBR, and TBR refer to false-claim and true-claim inference scores and belief ratings, respectively. Conditions are *NE* no-exposure; *CO* claim-only; *FCO* fact-check-only; *CFC* claim-plus-fact-check. ^a^indicates statistical significance (for secondary contrasts: after Holm-Bonferroni correction)

The effectiveness of correcting a previously encountered false claim was investigated by contrasting the claim-plus-fact-check (CFC *M* = 5.04, *SE* = 0.11) and claim-only conditions. The difference was non-significant, and so H3_FIS_ was rejected.

In order to test whether correcting previously presented false claims would reduce inference scores below baseline, the no-exposure condition was contrasted with the claim-plus-fact-check condition. The difference was clearly non-significant, so H4_FIS_ was also rejected.

##### False-claim belief ratings

**Fig. 4:**
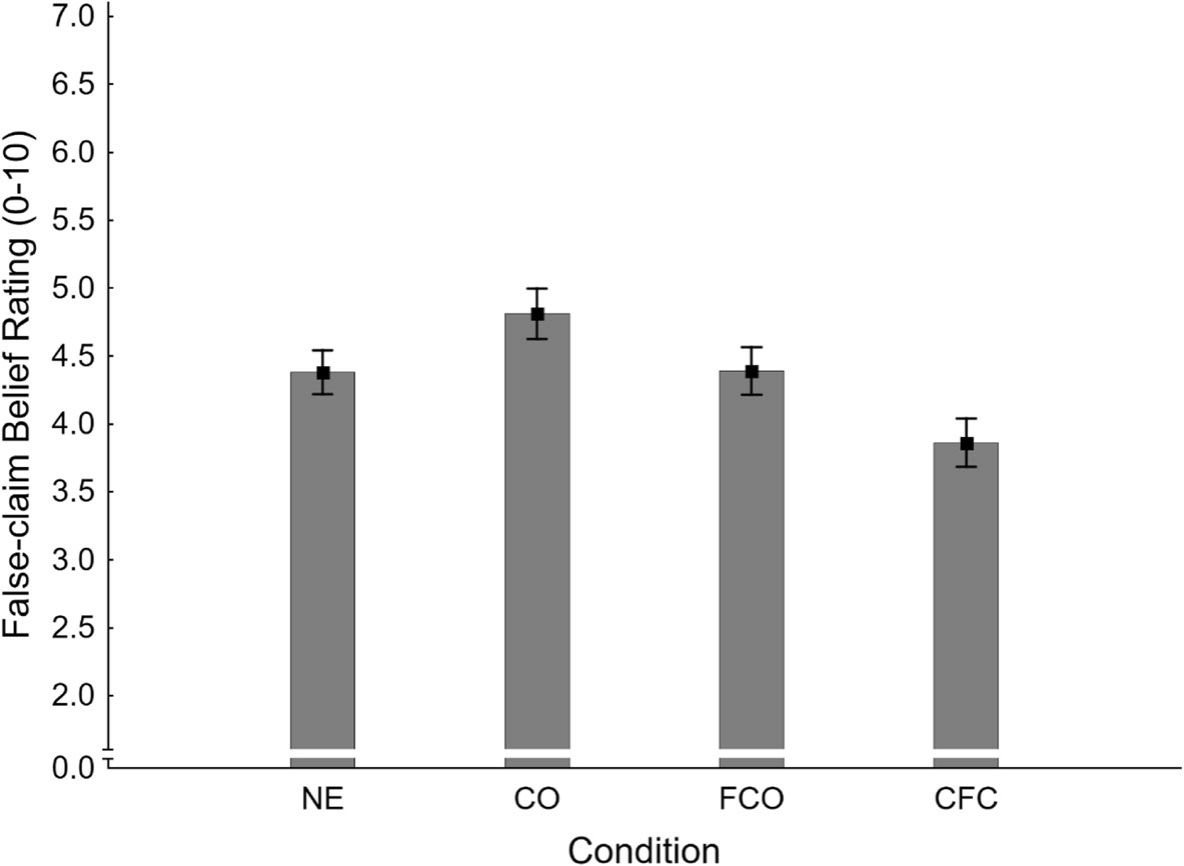
Mean false-claim belief ratings across conditions NE (no-exposure), CO (claim-only), FCO (fact-check-only), and CFC (claim-plus-fact-check) in Experiment 1. Error bars show standard errors of the mean

Mean false-claim belief ratings across conditions are shown in Fig. 4. A one-way ANOVA found a significant main effect of condition, *F*(3,367) = 4.65, *η*_*p*_^2^ = .037, *p* = .003. A series of four planned contrasts was then conducted, the results of which are reported in the second panel of Table 1.

In order to test for a familiarity backfire effect in belief ratings, the no-exposure condition (NE *M* = 4.38, *SE* = 0.16) was contrasted with the fact-check-only condition (FCO: *M* = 4.39, *SE* = 0.17). The difference was non-significant, and thus no additional evidence for familiarity backfire was obtained; H1_FBR_ was rejected.

To test for an illusory truth effect, we compared no-exposure to claim-only (CO *M* = 4.81, *SE* = 0.19) conditions. Belief ratings were numerically higher in the claim-only condition, but the difference was non-significant; H2_FBR_ was, therefore, rejected.

The effectiveness of corrections targeting a previously encountered false claim was tested by contrasting the claim-plus-fact-check (CFC *M* = 3.86, *SE* = 0.18) and claim-only conditions. Belief ratings were significantly lower in the claim-plus-fact-check condition, supporting H3_FBR_.

#### True claims

##### True-claim inference scores

**Fig. 5:**
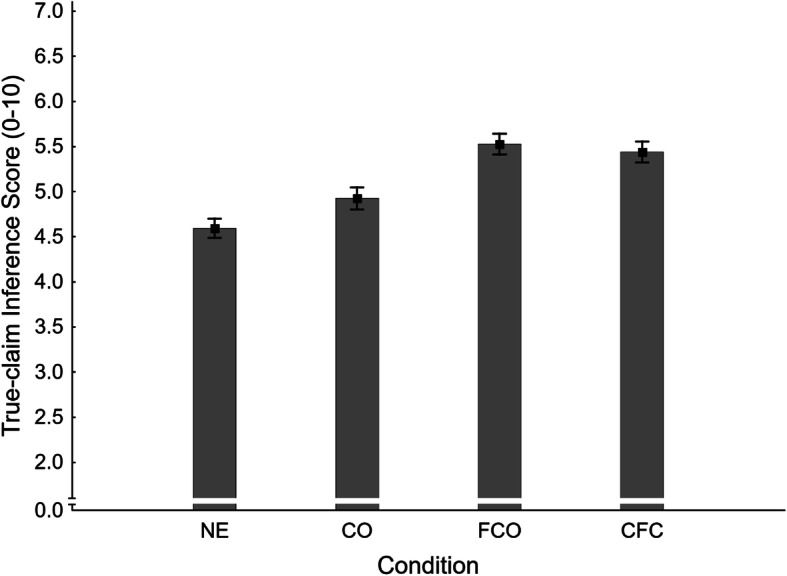
Mean true-claim inference scores across conditions NE (no-exposure), CO (claim-only), FCO (fact-check-only), and CFC (claim-plus-fact-check) in Experiment 1. Error bars show standard errors of the mean

Mean true-claim inference scores across conditions are shown in Fig. 5. A one-way ANOVA indicated a significant main effect of condition, *F*(3,367) = 15.92, *η*_*p*_^2^ = .115, *p* < .001. Three planned contrasts tested for specific condition differences. Results are reported in the third panel of Table 1.

To test if mere affirmations increased inference scores relative to baseline, we compared no-exposure (NE *M* = 4.59, *SE* = 0.11) and fact-check-only (FCO *M* = 5.53, *SE* = 0.11) conditions. This was a highly significant difference, so H1_TIS_ was supported.

The illusory truth effect was tested for by contrasting no-exposure and claim-only (CO *M* = 4.92, *SE* = 0.12) conditions. Inference scores were significantly greater in the claim-only condition; H2_TIS_ was, therefore, supported.

The effectiveness of fact-checks affirming previously encountered claims was examined by contrasting claim-plus-fact-check (CFC *M* = 5.44, *SE* = 0.12) and claim-only conditions. Inference scores were found to be significantly greater in the claim-plus-fact-check condition, so H3_TIS_ was also supported.

To test the effectiveness of correcting a previously presented false claim relative to baseline, the claim-plus-fact-check condition was compared to no-exposure control. Belief ratings were numerically lower in the claim-plus-fact-check condition, but the contrast was non-significant after correcting for multiple tests; H4_FBR_ was thus rejected.

##### True-claim belief ratings

**Fig. 6:**
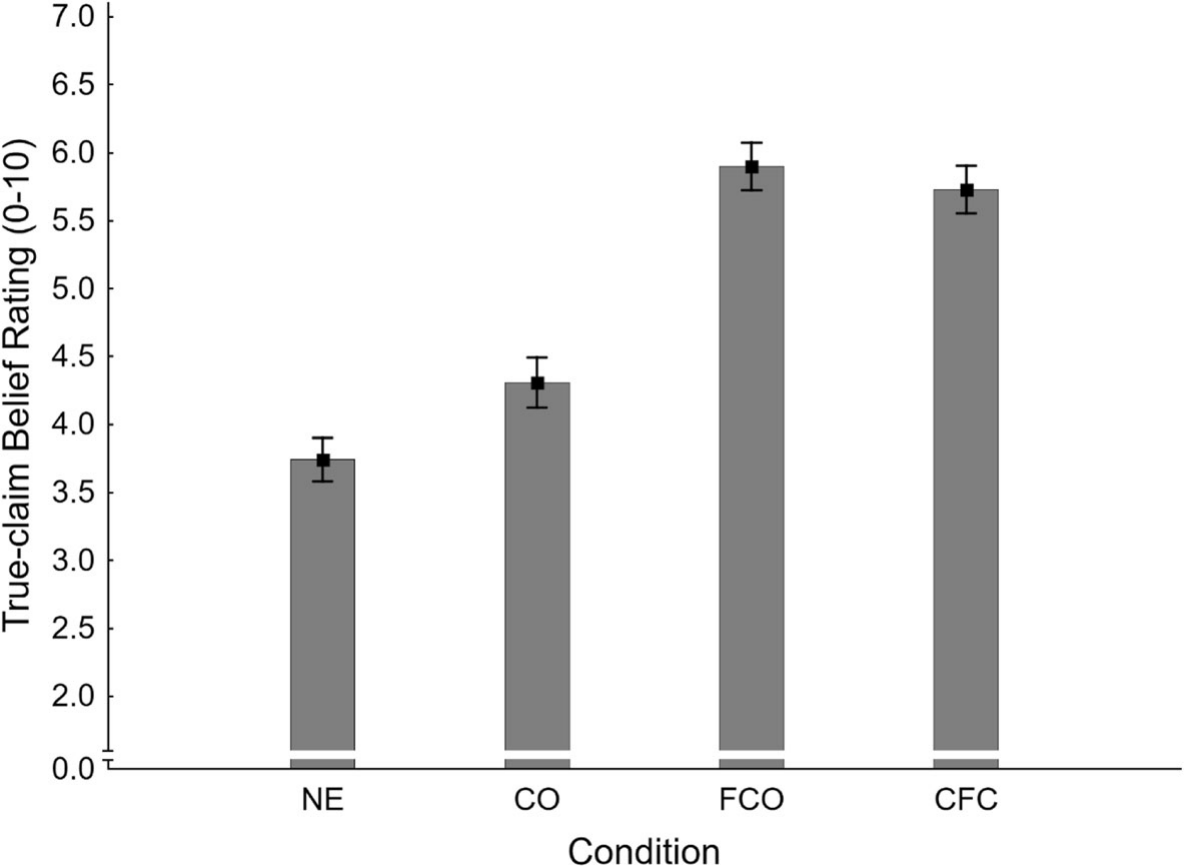
Mean true-claim belief ratings across conditions NE (no-exposure), CO (claim-only), FCO (fact-check-only), and CFC (claim-plus-fact-check) in Experiment 1. Error bars show standard errors of the mean

Mean true-claim belief ratings across conditions are shown in Fig. 6. A one-way ANOVA returned a significant main effect of condition, *F*(3,367) = 39.08, *η*_*p*_^2^ = .242, *p* < .001. Three planned contrasts were performed; results are presented in the fourth panel of Table 1.

To test the effectiveness of a mere affirmation relative to baseline, we compared the no-exposure condition (NE *M* = 3.74, *SE* = 0.16) with the fact-check-only condition (FCO: *M* = 5.90, *SE* = 0.17). The difference was found to be highly significant, supporting H1_TBR_.

To test for an illusory truth effect, we contrasted no-exposure and claim-only (CO *M* = 4.31, *SE* = 0.19) conditions. Belief ratings were significantly higher in the claim-only condition, providing evidence for an illusory truth effect and supporting H2_TBR_.

Finally, we contrasted the claim-plus-fact-check (CFC *M* = 5.73, *SE* = 0.18) and claim-only conditions to test whether an affirmation of a previously presented claim enhanced belief. Belief was higher in the claim-plus-fact-check condition, and so H3_TBR_ was supported.

### Discussion

Experiment 1 found evidence for a small familiarity backfire effect on inference scores, supporting Skurnik et al. (2005). After a 1-week study-test delay, participants who were exposed only to the corrective fact-check showed reasoning more in line with the false claim than participants never exposed to either the claim or the fact-check. This provides tentative evidence that corrections can backfire and ironically increase misinformed reasoning when they familiarize people with novel misinformation. However, no familiarity backfire effect was observed on direct belief ratings, suggesting that exposure to the previously corrected claim at test may have facilitated recollection of the correction. Given the small magnitude of the effect on inference scores, we aimed to replicate the result in Experiment 2 before drawing stronger conclusions; however, to foreshadow, the effect did not replicate.

Furthermore, Experiment 1 provided some additional evidence for illusory truth effects after just a single exposure (Begg et al., 1992; Dechêne et al., 2010; Pennycook et al., 2018): participants’ claim-congruent reasoning and beliefs were stronger for claims that they were previously exposed to, at least when the claims were actually true.

In general, it was found that fact-checks were effective when they targeted a claim that participants had already encountered before. Relative to the claim-only condition, the claim-plus-fact-check condition reduced false-claim beliefs and increased true-claim beliefs as well as true-claim-congruent reasoning (the reduction in false-claim inference scores was non-significant). These results replicate Ecker et al.’s (2020) finding that fact-checks tended to be more impactful if participants had previously been exposed to the relevant claim. The overall pattern also replicates Swire et al. (2017) in that affirmations tended to be more impactful than corrections, presumably because familiarity and recollection operate in unison for true claims (both driving acceptance) but stand in opposition with false claims (where claim familiarity will foster acceptance but correction recollection will drive rejection). However, correcting previously presented false claims did not reduce inference scores below the no-exposure baseline (the effect for belief ratings was marginal but non-significant). This contrasts to some extent with the findings of Ecker et al. (2020), although that study did not contrast no-exposure and claim-plus-fact-check conditions after a 1-week delay. The absence of a stronger reduction is, therefore, again best explained by the tension between familiarity and recollection processes, with the latter more strongly compromised by the substantial retention interval.

## Experiment 2

The aim of Experiment 2 was to replicate the familiarity backfire effect found in Experiment 1. Additionally, Experiment 2 manipulated retention interval, so the test was either immediate (henceforth indicated by lower-case i) or by 1 week as in Experiment 1 (indicated by lower-case d). The rationale for this was that a familiarity backfire effect should arise only with a delayed test, not an immediate test, when recollection of the correction will still be strong enough to avoid ironic correction effects. Experiment 2 therefore replicated exactly the four experimental conditions of Experiment 1, but added claim-only, fact-check-only, and claim-plus-fact-check conditions with immediate test; it thus had a between-subjects design with the sole factor of condition (NE; COi; FCOi; CFCi; COd; FCOd; CFCd).

The design and analysis plan for Experiment 2 were pre-registered (https://osf.io/69bq3/registrations). As in Experiment 1, the core hypothesis regarded the familiarity backfire effect; it was hypothesized that false-claim inference scores would be higher in the delayed fact-check-only condition relative to no-exposure control (H1_FISd_; NE < FCOd). A related secondary hypothesis was that in the immediate test, there should be no backfire and indeed a corrective effect (H1_FISi_; NE > FCOi).

Supplementary hypotheses included the supplementary hypotheses of Experiment 1 (we refrain from repeating these here, but they are specified again in Table 2); additional supplementary hypotheses were formulated regarding the effects of the delay manipulation on scores in the fact-check-only (H5) and claim-plus-fact-check (H6) conditions. It was assumed that significant forgetting would occur over time, implying that false-claim inference scores and belief ratings would be lower in the immediate fact-check-only (FCOi) and claim-plus-fact-check (CFCi) conditions than the respective delayed conditions (H5_FIS_ and H5_FBR_; see Table 2), and that true-claim inference scores and belief ratings would be higher in the immediate fact-check-only (FCOi) and claim-plus-fact-check (CFCi) conditions than the respective delayed conditions (H5_TIS_ and H5_TBR_; see Table 2).

### Method

#### Participants

A power analysis indicated that to detect an effect of the size observed in Experiment 1 (main effect of condition on false-claim inference scores, *η*_*p*_^2^ = .022) with *α* = 0.05 and 1 − *β* = 0.80 across the four replicated conditions would require a minimum sample size of *n* = 123 per condition. In Experiment 1, the lowest retention of any of the conditions was 81/110 = 73.63% (condition CO). It was thus decided to recruit *n* = 170 participants per condition in the delayed-test conditions and *n* = 130 participants in the immediate-test conditions and the no-exposure condition, in the hope of achieving a test-phase sample size of *n* ≈ 130 per condition (i.e., total *N* = 3 × 170 + 4 × 130 = 1030). Participants were US-based adult MTurk workers who had completed at least 5000 HITs with 97% + approval. Participants who had completed Experiment 1 were excluded from participation. The delayed-test conditions, the immediate-test conditions, and the no-exposure condition were again run separately due to differences in instructions and reimbursements, with random condition assignment in the delayed and immediate surveys. The immediate-test and no-exposure conditions were run concurrently with the delayed test; participants were not able to complete more than one condition.

A subset of 509 participants was randomly assigned to one of the three delayed-test conditions, with the constraint of approximately equal cell sizes. The retention rate between study and test was approximately 84%, with 427 participants returning for the test phase. An additional 521 participants completed the immediate-test and NE conditions. Nine participants were excluded based on a-priori criteria (see the “Results” section for details). The final sample size for analysis was thus *N* = 939 (condition NE *n* = 128; COi *n* = 129; FCOi *n* = 129; CFCi *n* = 129; COd *n* = 140; FCOd *n* = 144; CFCd *n* = 140; age range 20–81 years; *M*_age_ *=* 41.35; *SD*_age_ = 11.97; 469 male, 467 female, and 3 participants of undisclosed gender). Participants were paid US$0.40 for the study phase and US$0.60 for the test phase.

#### Materials

Claims, measures, and procedure were identical to Experiment 1, except that Experiment 2 also contained an immediate test, where participants just completed a 1-min word puzzle between study and test.

### Results

Before analysis, we applied a set of a-priori (pre-registered) exclusion criteria. Two criteria were not met by any participants, including English proficiency self-rated as “poor,” and self-reported lack of effort. Uniform responding and erratic responding were identified as in Experiment 1, which led to the exclusion of *n* = 5 and *n* = 4 participants, respectively. Inference and belief scores were calculated as in Experiment 1.

#### False claims

##### False-claim inference scores

**Fig. 7:**
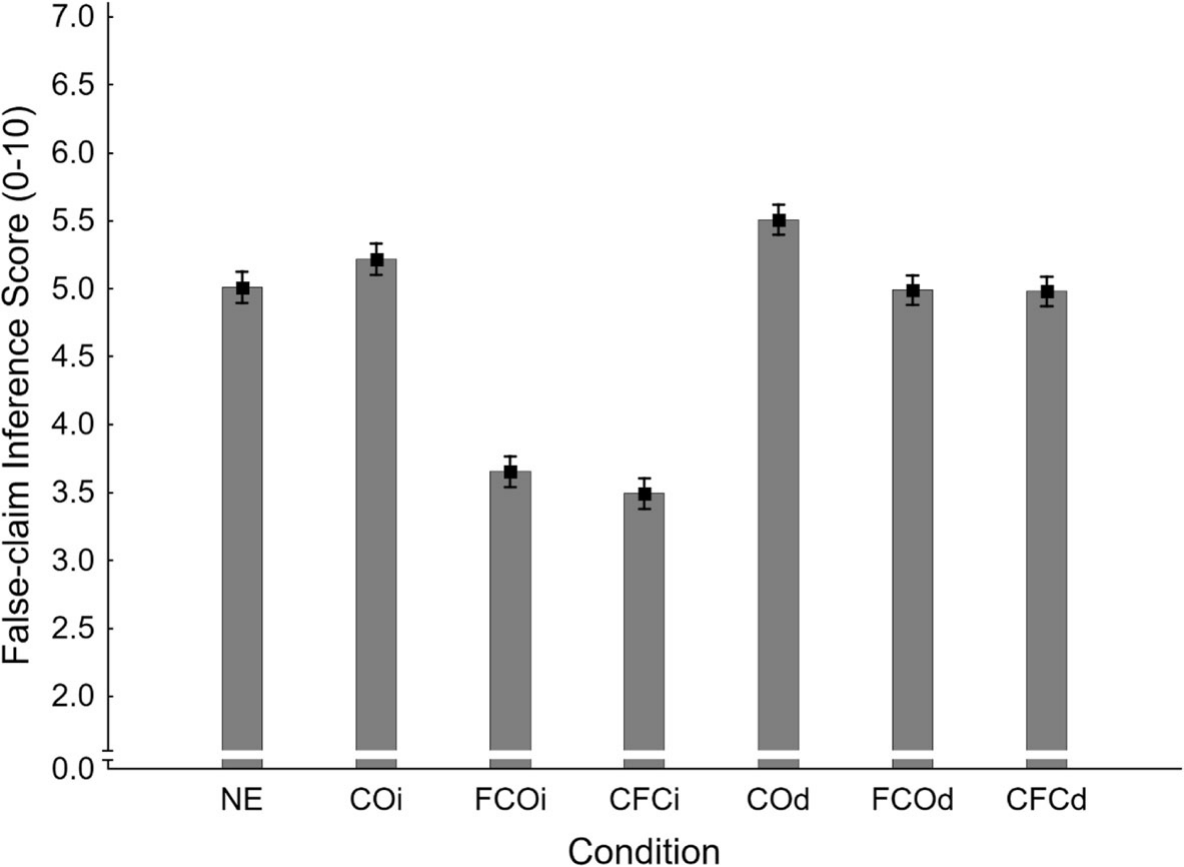
Mean false-claim inference scores across conditions NE (no-exposure), COi/d (claim-only, immediate/delayed test), FCOi/d (fact-check-only, immediate/delayed test), and CFCi/d (claim-plus-fact-check, immediate/delayed test) in Experiment 2. Error bars show standard errors of the mean

Mean false-claim inference scores across conditions are shown in Fig. 7. A one-way ANOVA revealed a significant main effect of condition, *F*(6,932) = 48.66, *η*_*p*_^*2*^ = .239, *p* < .001. To test the primary hypothesis that corrections of novel myths would produce a familiarity backfire effect, a planned contrast compared no-exposure (NE *M* = 5.01, *SE* = 0.11) and delayed fact-check-only (FCOd *M* = 4.99, *SE* = 0.11) conditions. This was clearly non-significant, *F*(1,932) = 0.02, *η*_*p*_^2^ < .001, *p* = .895. Thus, no familiarity backfire effect was observed, and H1_FISd_ was not supported. However, the inference score in the immediate fact-check-only condition (FCOi *M* = 3.65, *SE* = 0.11) was significantly lower than the no-exposure control, supporting secondary hypothesis H1_FISi_.

Next, the supplementary planned contrasts were conducted on false-claim inference scores. Results are reported in the first panel of Table 2 (together with the primary contrast). To summarize, we found evidence of an illusory truth effect in the delayed (COd *M* = 5.51, *SE* = 0.11) but not the immediate test (COi *M* = 5.22, *SE* = 0.11), rejecting H2_FISi_ and supporting H2_FISd_. Corrections of previously presented false claims (CFCi *M* = 3.49, *SE* = 0.11; CFCd *M* = 4.98, *SE* = 0.11) were found effective relative to the claim-only condition at both delays (supporting H3_FISi_ and H3_FISd_). However, compared against the no-exposure baseline, corrections of previously presented false claims were effective immediately but not after a delay (supporting H4_FISi_ and rejecting H4_FISd_). As expected, the delay had a significant impact on correction effectiveness in both fact-check-only and claim-plus-fact-check conditions (supporting H5_FIS_ and H6_FIS_).

**Table 2:** Contrasts run in Experiment 2

dV/hypothesis	Effect tested	*F*(1,932)	*P*
False-claim inference scores			
H1_FISi_: NE > FCOi	Effect of correction vs. baseline	70.33	< .001^a^
H2_FISi_: NE < COi	Illusory truth effect	1.63	.202
H3_FISi_: CFCi < COi	Effect of claim+correction vs. claim-only	114.14	< .001^a^
H4_FISi_: NE > CFCi	Effect of claim+correction vs. baseline	88.08	< .001^a^
**H1**_**FISd**_**: NE < FCOd**	**Familiarity backfire effect**	**0.02**	**.895**
H2_FISd_: NE < COd	Illusory truth effect	9.89	.002^a^
H3_FISd_: CFCd < COd	Effect of claim+correction vs. claim-only	11.64	< .001^a^
H4_FISd_: NE > CFCd	Effect of claim+correction vs. baseline	0.04	.850
H5_FIS_: FCOi < FCOd	Delay effect on correction	72.21	< .001^a^
H6_FIS_: CFCi < CFCd	Delay effect on claim+correction	88.43	< .001^a^
False-claim belief ratings			
H1_FBRi_: NE > FCOi	Effect of correction vs. baseline	83.13	< .001^a^
H2_FBRi_: NE < COi	Illusory truth effect	3.75	.053^b^
H3_FBRi_: CFCi < COi	Effect of claim+correction vs. claim-only	87.36	< .001^a^
H4_FBRi_: NE > CFCi	Effect of claim+correction vs. baseline	126.87	< .001^a^
H1_FBRd_: NE < FCOd	Familiarity backfire effect	2.02	.155
H2_FBRd_: NE < COd	Illusory truth effect	14.09	< .001^a^
H3_FBRd_: CFCd < COd	Effect of claim+correction vs. claim-only	37.91	< .001^a^
H4_FBRd_: NE > CFCd	Effect of claim+correction vs. baseline	5.12	.024
H5_FBR_: FCOi < FCOd	Delay effect on correction	63.33	< .001^a^
H6_FBR_: CFCi < CFCd	Delay effect on claim+correction	85.50	< .001^a^
True-claim inference scores			
H1_TISi_: NE < FCOi	Effect of affirmation vs. baseline	148.92	< .001^a^
H2_TISi_: NE < COi	Illusory truth effect	2.21	.137
H3_TISi_: CFCi > COi	Effect of claim+affirmation vs. claim-only	121.24	< .001^a^
H1_TISd_: NE < FCOd	Effect of affirmation vs. baseline	18.24	< .001^a^
H2_TISd_: NE < COd	Illusory truth effect	0.19	.666
H3_TISd_: CFCd > COd	Effect of claim+affirmation vs. claim-only	15.07	< .001^a^
H5_TIS_: FCOi > FCOd	Delay effect on affirmation	23.51	< .001^a^
H6_TIS_: CFCi > CFCd	Delay effect on claim+affirmation	72.57	< .001^a^
True-claim belief ratings			
H1_TBRi_: NE < FCOi	Effect of affirmation vs. baseline	45.71	< .001^a^
H2_TBRi_: NE < COi	Illusory truth effect	3.79	.052^b^
H3_TBRi_: CFCi > COi	Effect of claim+affirmation vs. claim-only	108.75	< .001^a^
H1_TBRd_: NE < FCOd	Effect of affirmation vs. baseline	57.16	< .001^a^
H2_TBRd_: NE < COd	Illusory truth effect	6.49	.011^a^
H3_TBRd_: CFC_D_ > COd	Effect of claim+affirmation vs. claim-only	25.31	< .001^a^
H5_TBR_: FCOi > FCOd	Delay effect on affirmation	0.38	.536
H6_TBR_: CFCi > CFCd	Delay effect on claim+affirmation	1.37	.243

*Note.* Hypotheses are numbered H1–6 (primary hypothesis in bold; see text for details); subscripts FISi/d, TISi/d, FBRi/d, and TISi/d refer to false-claim and true-claim inference scores and belief ratings in immediate and delayed tests, respectively. Conditions are *NE* no-exposure; *COi/d* claim-only with immediate/delayed test; *FCOi/d* fact-check-only with immediate/delayed test; *CFCi/d* claim-plus-fact-check with immediate/delayed test. ^a^indicates statistical significance after Holm-Bonferroni correction. ^b^indicates an effect in the opposite of hypothesized direction

##### False-claim belief ratings

**Fig. 8:**
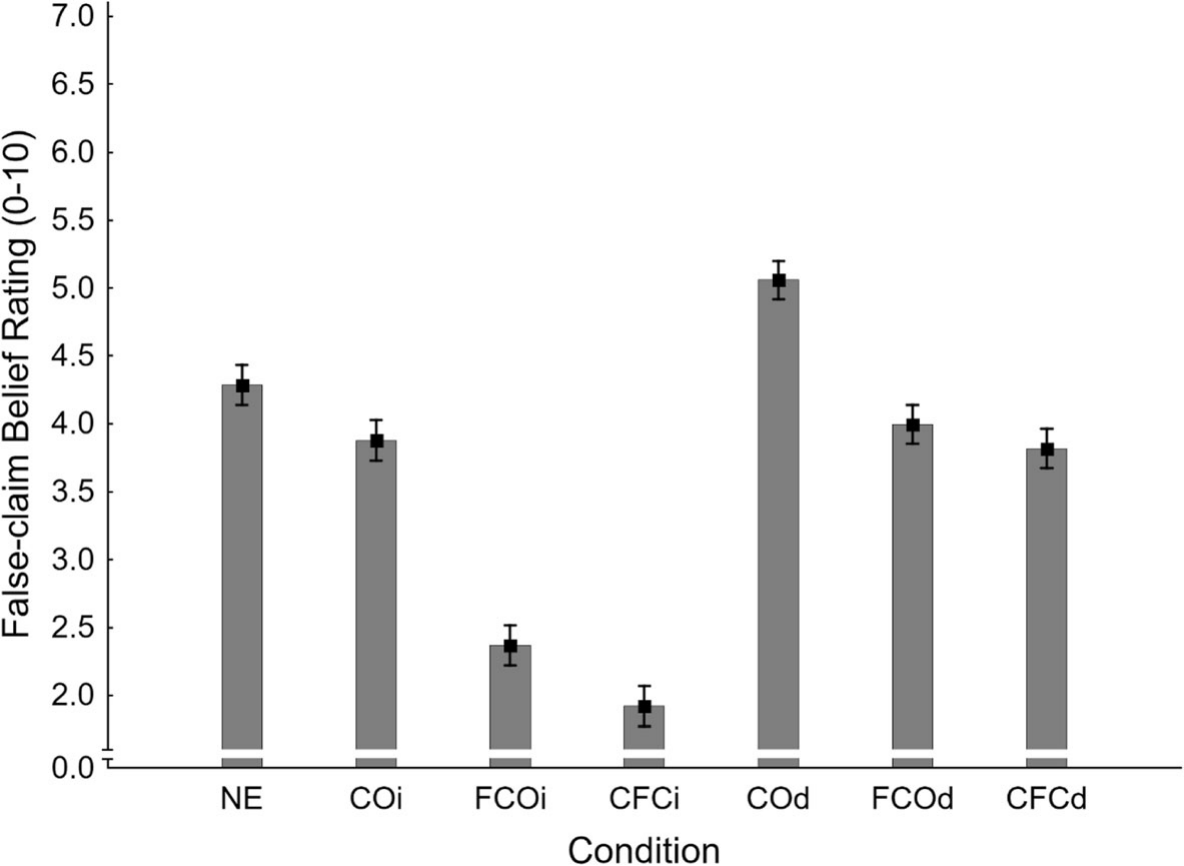
Mean false-claim belief ratings across conditions NE (no-exposure), COi/d (claim-only, immediate/delayed test), FCOi/d (fact-check-only, immediate/delayed test), and CFCi/d (claim-plus-fact-check, immediate/delayed test) in Experiment 2. Error bars show standard errors of the mean

Mean false-claim belief ratings across conditions are shown in Fig. 8. A one-way ANOVA revealed a significant main effect of condition, *F*(6,932) = 56.14, *η*_*p*_^2^ = .265, *p* < .001. Planned contrasts were run to test specific hypotheses; results are provided in the second panel of Table 2.

There was no evidence of familiarity backfire in belief ratings, as the delayed fact-check-only condition (FCOd *M* = 3.99, *SE* = 0.14) did not differ significantly from no-exposure control (NE *M* = 4.29, *SE* = 0.15); H1_FBRd_ was thus rejected. However, a mere correction was effective in the immediate test (FCOi *M* = 2.37, *SE* = 0.15), supporting H1_FBRi_. There was mixed evidence regarding illusory truth effects, with no-exposure differing significantly from the claim-only condition in the delayed test (COd *M* = 5.06, *SE* = 0.14) but not the immediate test (COi *M* = 3.88, *SE* = 0.15), supporting H2_FBRd_ but rejecting H2_FBRi_. Corrections of previously presented false claims (CFCi *M* = 1.92, *SE* = 0.15; CFCd *M* = 3.82, *SE* = 0.14) were found effective relative to the claim-only condition at both delays (supporting H3_FBRi_ and H3_FBRd_). However, mirroring the inference-score results, compared against the no-exposure baseline, corrections of previously presented false claims were effective immediately but not after a delay (supporting H4_FBRi_ and rejecting H4_FBRd_). Delay again had a significant impact on correction effectiveness in both fact-check-only and claim-plus-fact-check conditions (supporting H5_FBR_ and H6_FBR_).

#### True claims

##### True-claim inference scores

**Fig. 9:**
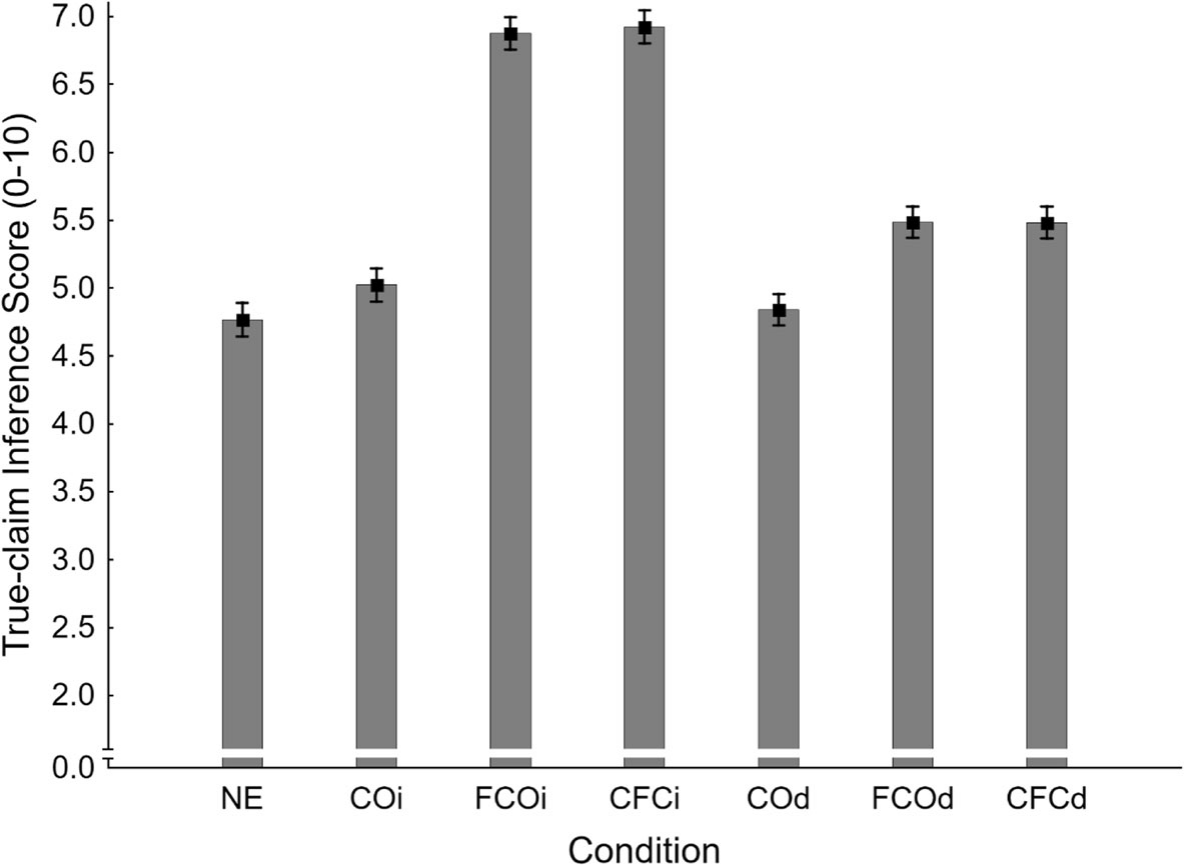
Mean true-claim inference scores across conditions NE (no-exposure), COi/d (claim-only, immediate/delayed test), FCOi/d (fact-check-only, immediate/delayed test), and CFCi/d (claim-plus-fact-check, immediate/delayed test) in Experiment 2. Error bars show standard errors of the mean

Mean true-claim inference scores across conditions are shown in Fig. 9. A one-way ANOVA indicated a significant main effect of condition, *F*(6,932) = 56.62, *η*_*p*_^2^ = .267, *p* < .001. Planned contrasts tested for specific condition differences; results are reported in the third panel of Table 2.

It was found that a mere affirmation increased inference scores relative to the no-exposure baseline (NE *M* = 4.77, *SE* = 0.12) in both immediate (FCOi *M* = 6.87, *SE* = 0.12) and delayed (FCOd *M* = 5.48, *SE* = 0.12) tests, supporting H1_TISi_ and H1_TISd_. There was no evidence for illusory truth effects, with no significant difference between claim-only and no-exposure conditions in either the immediate (COi *M* = 5.02, *SE* = 0.12) or delayed (COd *M* = 4.84, *SE* = 0.12) test; H2_TISi_ and H2_TISd_ were thus rejected. Affirmations of previously presented true claims (CFCi *M* = 6.92, *SE* = 0.12; CFCd *M* = 5.48, *SE* = 0.12) were found effective relative to the claim-only condition at both delays (supporting H3_TISi_ and H3_TISd_). Again, delay had a significant impact on affirmation effectiveness in both fact-check-only and claim-plus-fact-check conditions (supporting H5_TIS_ and H6_TIS_).

##### True-claim belief ratings

**Fig. 10:**
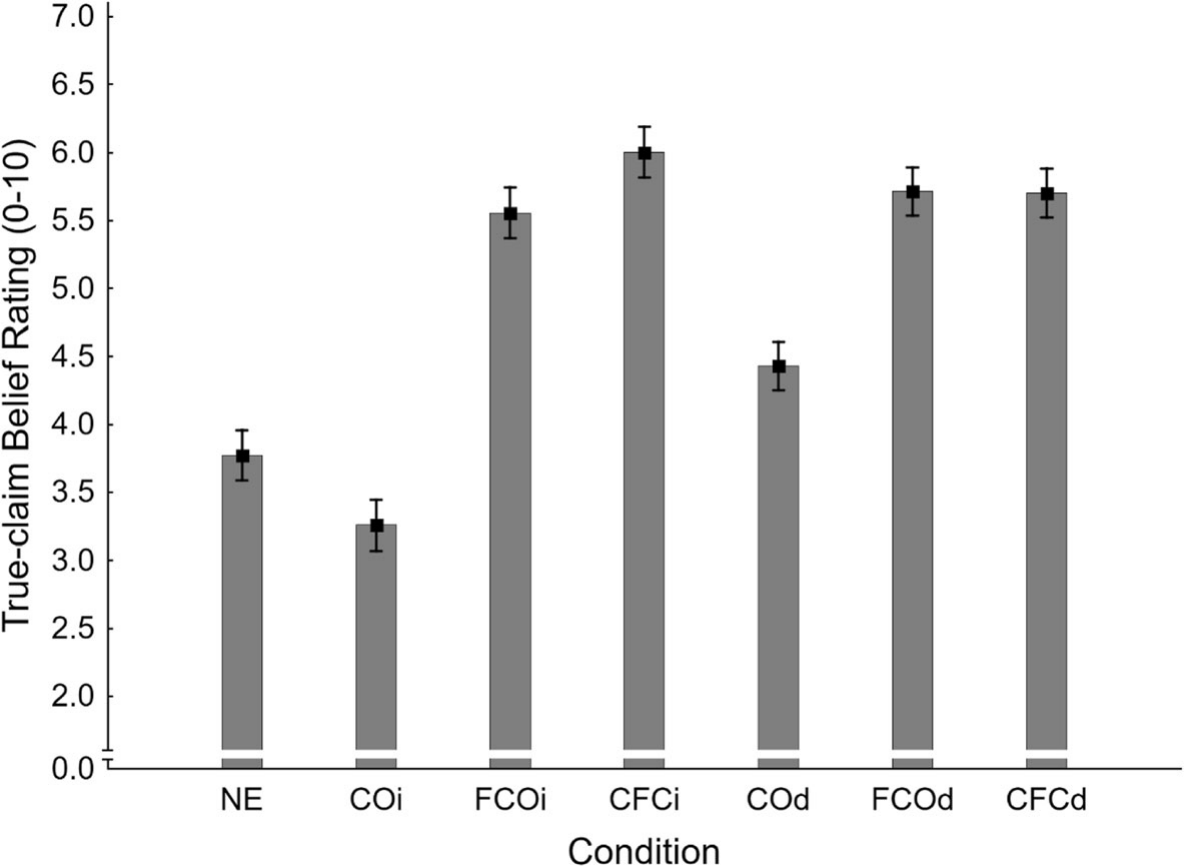
Mean true-claim belief ratings across conditions NE (no-exposure), COi/d (claim-only, immediate/delayed test), FCOi/d (fact-check-only, immediate/delayed test), and CFCi/d (claim-plus-fact-check, immediate/delayed test) in Experiment 2. Error bars show standard errors of the mean

Mean true-claim belief ratings across conditions are shown in Fig. 10. A one-way ANOVA returned a significant main effect of condition, *F*(6,932) = 34.98, *η*_*p*_^2^ = .184, *p* < .001. Planned contrasts tested for specific condition differences; results are reported in the fourth panel of Table 2.

It was found that a mere affirmation increased belief ratings relative to the no-exposure baseline (NE *M* = 3.77, *SE* = 0.19) in both immediate (FCOi *M* = 5.56, *SE* = 0.19) and delayed (FCOd *M* = 5.71, *SE* = 0.18) tests, supporting H1_TBRi_ and H1_TBRd_. There was mixed evidence for illusory truth effects, with a significant difference between claim-only and no-exposure conditions in the delayed (COd *M* = 4.43, *SE* = 0.18) but not the immediate (COi *M* = 3.26, *SE* = 0.19) test, supporting H2_TBRd_ and rejecting H2_TBRi_. Affirmations of previously presented true claims (CFCi *M* = 6.00, *SE* = 0.19; CFCd *M* = 5.70, *SE* = 0.18) were found effective relative to the claim-only condition at both delays (supporting H3_TBRi_ and H3_TBRd_). In contrast to the inference scores, delay had no significant impact on affirmation effectiveness in fact-check-only and claim-plus-fact-check conditions (rejecting H5_TBR_ and H6_TBR_).

### Discussion

The primary aim of Experiment 2 was to replicate the familiarity backfire effect observed in Experiment 1. The effect did not replicate; there was no evidence for familiarity backfire in either the false-claim inference scores or the false-claim belief scores. This is consonant with the results that Ecker et al. (2020) obtained with non-novel claims, and suggests that the familiarity boost effected by exposure to a false claim within a correction may be sufficient to offset the corrective effect of a mere fact-check after a 1-week delay (thus resulting in the observed null effect), but not sufficient to cause ironic misconception-strengthening effects.

Evidence for illusory truth effects was again mixed: false-claim inference scores and belief ratings, as well as true-claim belief ratings, were greater in the claim-only condition compared to the no-exposure baseline in a delayed test. This stands in contrast to Experiment 1, where illusory truth effects were found only for true claims. Given that participants were unable to reliably differentiate between true and false claims prior to fact-checks being provided, we suspect that the best explanation for the overall pattern is that illusory truth effects after a single exposure are small, and whether or not a statistically significant effect is obtained is partially down to random variation. There were no significant illusory truth effects in the immediate test, suggesting that illusory truth effects may be delay-dependent and thus occur only if memory is relatively more reliant on familiarity.

As in Experiment 1, fact-checks were generally effective when they targeted a claim that participants had already encountered before. Relative to the claim-only condition, the claim-plus-fact-check condition reduced false-claim beliefs and false-claim-congruent reasoning and increased true-claim beliefs and true-claim-congruent reasoning across both retention intervals. This again replicates the findings of Ecker et al. (2020) that fact-checks are more impactful if participants had previously been exposed to the relevant claim. However, replicating Experiment 1, correcting previously presented false claims did not reduce inference scores or belief ratings below the no-exposure baseline after a delay. This is again best explained by the fact that familiarity and recollection processes stand in opposition when it comes to delayed appraisals of corrected false claims. Additional support for this theoretical notion comes from the pattern of delay effects observed: while both fact-check-only and claim-plus-fact-check corrections were much less effective at reducing false-claim belief and false-claim-congruent reasoning after a longer delay, with true-claim affirmations there were only delay effects on inference scores. No delay effect was observed for true-claim belief ratings, meaning that the effect of affirmations did not wear off significantly over the course of a week. This mirrors the findings of Swire et al. (2017), who proposed the notion that correction effects are less sustained than affirmation effects due to the influence of claim familiarity. The post-affirmation reduction in true-claim inference scores after a delay is presumably due to the fact that the inference questions did not contain strong retrieval cues.

Given that Experiments 1 and 2 yielded contradictory evidence regarding the presence of familiarity backfire effects, we conducted Experiment 3. Experiment 3 was a replication of the no-exposure and delayed fact-check-only conditions of Experiments 1 and 2, with an additional manipulation of cognitive load during encoding. The rationale for this manipulation was that familiarity backfire effects should be more likely under cognitive-load conditions.

## Experiment 3

Experiment 3 was conducted with the aim of replicating the familiarity backfire effect observed in Experiment 1 but clearly absent in Experiment 2. It implemented only the two conditions of main interest, viz. the no-exposure and delayed fact-check-only conditions. Additionally, cognitive load was manipulated (low vs. high, henceforth indicated as l- and l+). Cognitive load is induced by the division of attention between two demanding tasks; it is known to impair memory (e.g., Craik, Govoni, Naveh-Benjamin, & Anderson, 1996), and, in particular, more strategic memory processes rather than more automatic processes such as familiarity (e.g., Hicks & Marsh, 2000). Cognitive load may also specifically impair the processing of corrections (Ecker et al., 2010) such that the primary effect of a correction may be to boost the familiarity of the retracted claim.

The design and the analysis plan for Experiment 3 were pre-registered (https://osf.io/69bq3/registrations). As in Experiments 1 and 2, the core hypothesis pertained to the familiarity backfire effect; it was hypothesized that false-claim inference scores would be higher in the delayed fact-check-only condition under high load than no-exposure control (H1_FISl+_; NE < FCOl+). We also hypothesized that familiarity backfire would occur without load (H1_FISl-_; NE < FCOl-), as in Experiment 1, even though based on Experiment 2 we did not expect to support this hypothesis.

Supplementary hypotheses included some of the supplementary hypotheses of Experiments 1 and 2; these are not repeated here but specified again in Table 3. Additional supplementary hypotheses were formulated regarding the effects of the cognitive-load manipulation on scores in the fact-check-only conditions. It was assumed that load would reduce correction effects. We therefore expected that false-claim inference scores and belief ratings would be greater in the load condition than the no-load condition (i.e., FCOl+ > FCOl-; H7_FIS_ and H7_FBR_, respectively; see Table 3), while true-claim inference scores and belief ratings would be greater in the no-load condition than the load condition (FCOl+ < FCOl-; H7_TIS_ and H7_TBR_, respectively; see Table 3).

### Method

#### Participants

Participants were US-based adult MTurk workers who had completed at least 5000 HITs with 97% + approval. Participants who had completed Experiment 1 or 2 were excluded from participation. The (two-phase) fact-check-only conditions were again run separately from the no-exposure condition, with random load-condition assignment within the fact-check-only conditions. The no-exposure condition was run concurrently with the delayed fact-check-only test; participants were not able to complete more than one condition.

Sampling decisions were guided by the power analysis presented in Experiment 2. A total of 400 participants were randomly assigned to one of the two fact-check-only conditions, with the constraint of approximately equal cell sizes. Failure to complete the secondary task above chance level led to the exclusion of *n* = 17 participants from the test phase. The retention rate between study and test was approximately 68%, with 260 participants returning for the test phase. An additional 151 participants completed the no-exposure condition. Three participants were excluded based on a-priori criteria (see the “Results” section for details). The final sample size for analysis was thus *N* = 408 (condition NE *n* = 150; FCOl- *n* = 128; FCOl+ *n* = 130; age range 20–74 years; *M*_age_ *=* 40.86; *SD*_age_ = 12.10; 180 male, 227 female, and 1 participant of undisclosed gender). Participants were paid US$0.40 for the study phase and US$0.60 for the test phase.

#### Materials

**Fig. 11:**
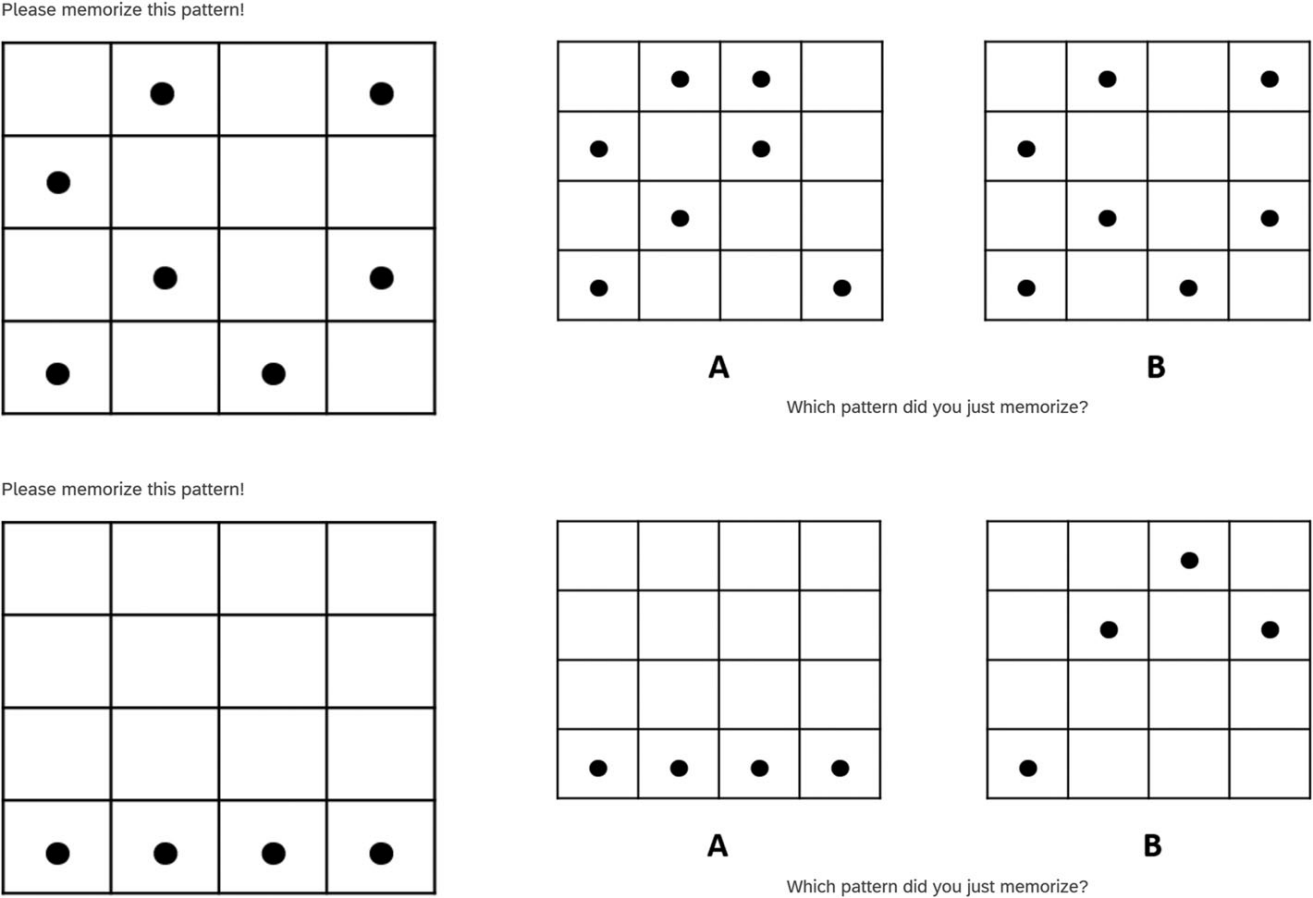
Example study and test patterns in fact-check-only conditions with high load (FCOl+; top) and low load (FCOl-; bottom)

Claims, measures, and procedure were identical to Experiment 1, with the exception of the secondary task—a dot-pattern-recognition task—used to manipulate cognitive load (following de Neys & Schaeken, 2007). Participants were presented with a dot matrix preceding each fact-check (2-s presentation time) and had to perform a 2AFC recognition test immediately after reading the fact-check. The to-be-remembered pattern was complex in the FCOl+ condition (seven dots in random locations, with no more than two (three) dots in any vertical/horizontal (diagonal) line; two to four dots overlap between the two test alternatives) but trivial in the FCOl- condition (four dots in a vertical/horizontal line; four random positions in test lure; see Fig. 11). Above-chance performance was defined as at least 8 out of 12 correct (cumulative probability when guessing *p* = .194).

### Results

Before analysis, we applied the same pre-registered exclusion criteria as in Experiments 1 and 2. The criterion of “poor” English proficiency was not met by any participant, but *n* = 1 participant was excluded due to self-reported lack of effort. Uniform and erratic responding each led to the exclusion of *n* = 1 participant. Inference and belief scores were calculated as in Experiments 1 and 2.

#### False-claim inference scores

**Fig. 12:**
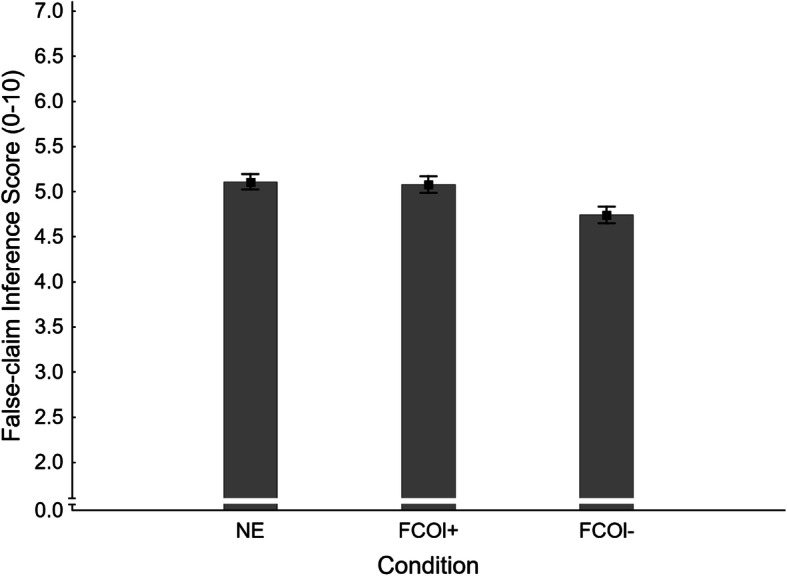
Mean false-claim inference scores across conditions NE (no-exposure) and FCOl± (fact-check-only, with/without cognitive load) in Experiment 3. Error bars show standard errors of the mean

Mean false-claim inference scores across conditions are shown in Fig. 12. A one-way ANOVA revealed a significant main effect of condition, *F*(2,405) = 5.00, *η*_*p*_^2^ = .024, *p* = .007. To test the primary hypothesis that corrections of novel myths would produce a familiarity backfire effect, a planned contrast compared the no-exposure condition (NE *M* = 5.11, *SE* = 0.09) with the fact-check-only condition with load (FCOl+ *M* = 5.08, *SE* = 0.09). This was clearly non-significant, *F*(1,405) = 0.06, *η*_*p*_^2^ < .001, *p* = .810. We also contrasted the no-exposure condition with the fact-check-only condition with no load (FCOl- *M* = 4.74, *SE* = 0.09), which mirrors the test for familiarity backfire in Experiments 1 and 2. This was significant, *F*(1,405) = 8.45, *η*_*p*_^2^ = .020, *p* = .004, but constituted a *corrective* effect (i.e., NE > FCOl-). Thus, no familiarity backfire effect was observed, and H1_FISl+_ and H1_FISl-_ were rejected. A supplementary planned contrast found a significant effect of cognitive load, supporting H7_FIS_ (see top panel of Table 3).

**Table 3:** Contrasts run in Experiment 3

dV/hypothesis	Effect tested	*F*(1,405)	*P*
False-claim inference scores			
**H1**_**FISl+**_**: NE < FCOl+**	**Familiarity backfire effect**	**0.06**	**.810**
**H1**_**FISl-**_**: NE < FCOl-**	**Familiarity backfire effect**	**8.45**	**.004**^**ab**^
H7_FIS_: FCOl- < FCOl+	Load effect on correction	6.65	.010^a^
False-claim belief ratings			
H1_FBRl-_: NE < FCOl-	Familiarity backfire effect	6.40	.012^ab^
H1_FBRl+_: NE < FCOl+	Familiarity backfire effect	3.39	.066^b^
H7_FBR_: FCOl- < FCOl+	Load effect on correction	0.45	.501
True-claim inference scores			
H1_TISl-_: NE < FCOl-	Effect of affirmation vs. baseline	19.21	< .001^a^
H1_TISl+_: NE < FCOl+	Effect of affirmation vs. baseline	15.69	< .001^a^
H7_TIS_: FCOl- > FCOl+	Load effect on affirmation	0.18	.671
True-claim belief ratings			
H1_TBRl-_: NE < FCOl-	Effect of affirmation vs. baseline	40.61	< .001^a^
H1_TBRl+_: NE < FCOl+	Effect of affirmation vs. baseline	22.32	< .001^a^
H7_TBR_: FCOl- > FCOl+	Load effect on affirmation	2.60	.108

*Note.* Hypotheses are numbered H1 and H7 (primary hypotheses in bold; see text for details); subscripts FIS, TIS, FBR, and TBR refer to false-claim and true-claim inference scores and belief ratings, respectively; no-load and load conditions are indicated by l- and l+. Conditions are *NE* no-exposure; *FCOl±* fact-check-only with no load or with load. ^a^indicates statistical significance (for secondary contrasts: after Holm-Bonferroni correction). ^b^indicates effect in the opposite of hypothesized direction

#### False-claim belief ratings

**Fig. 13:**
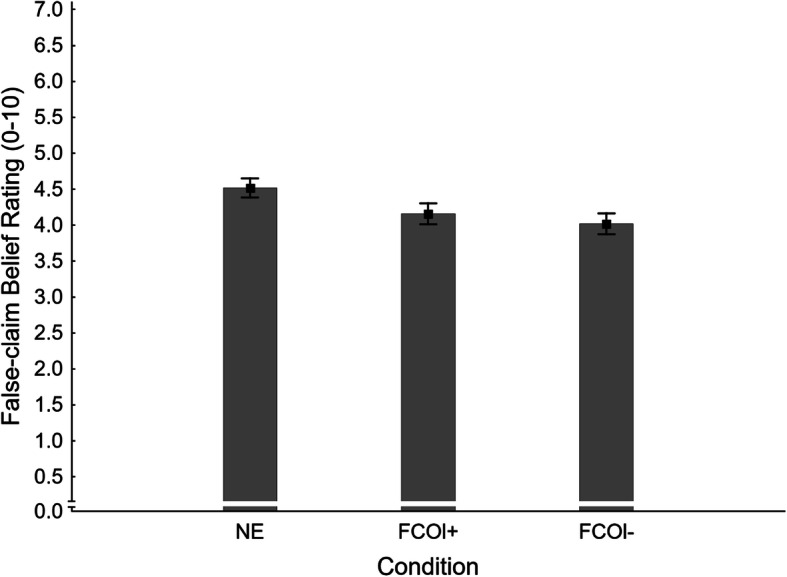
Mean false-claim belief ratings across conditions NE (no-exposure) and FCOl± (fact-check-only, with/without cognitive load) in Experiment 3. Error bars show standard errors of the mean

Mean false-claim belief ratings across conditions are shown in Fig. 13. A one-way ANOVA revealed a significant main effect of condition, *F*(2,405) = 3.49, *η*_*p*_^2^ = .017, *p* = .032. Planned contrasts were run to test specific hypotheses; results are provided in the second panel of Table 3.

It was found that a mere correction with no load at encoding (FCOl- *M* = 4.02, *SE* = 0.14) reduced false-claim belief relative to no-exposure control (NE *M* = 4.52, *SE* = 0.13); this rejects familiarity backfire hypothesis H1_FBRl-_. The fact-check-only condition with load (FCOl+ *M* = 4.16, *SE* = 0.14) did not differ significantly from either of the two other conditions; this rejects H1_FBRl+_ and H7_FBR_.

#### True-claim inference scores

**Fig. 14:**
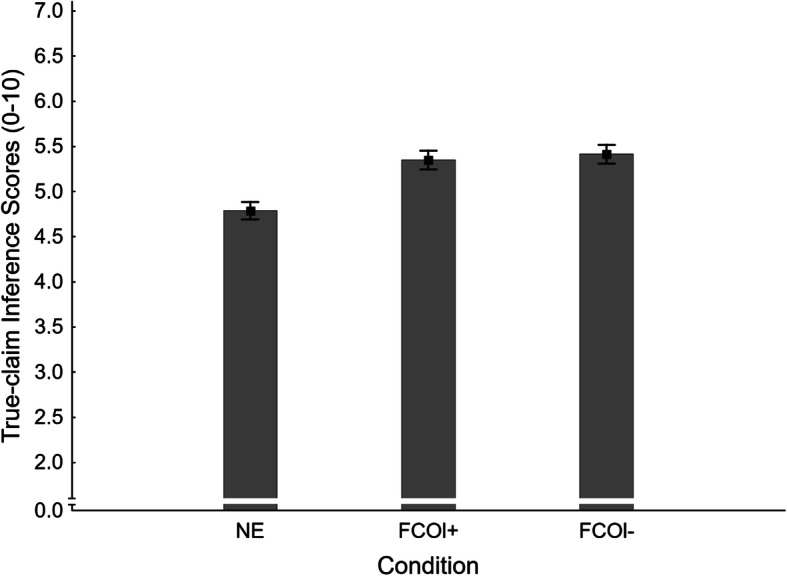
Mean true-claim inference scores across conditions NE (no-exposure) and FCOl± (fact-check-only, with/without cognitive load) in Experiment 3. Error bars show standard errors of the mean

Mean true-claim inference scores across conditions are shown in Fig. 14. A one-way ANOVA indicated a significant main effect of condition, *F*(2,405) = 11.99, *η*_*p*_^2^ = .056, *p* < .001. Planned contrasts tested for specific condition differences; results are reported in the third panel of Table 3. It was found that affirmations were equally effective across load conditions (NE *M* = 4.79, *SE* = 0.10; FCOl- *M* = 5.41, *SE* = 0.10; FCOl+ *M* = 5.35, *SE* = 0.10); this supports H1_TISl-_ and H1_TISl+_, and rejects H7_TIS_.

#### True-claim belief ratings

**Fig. 15:**
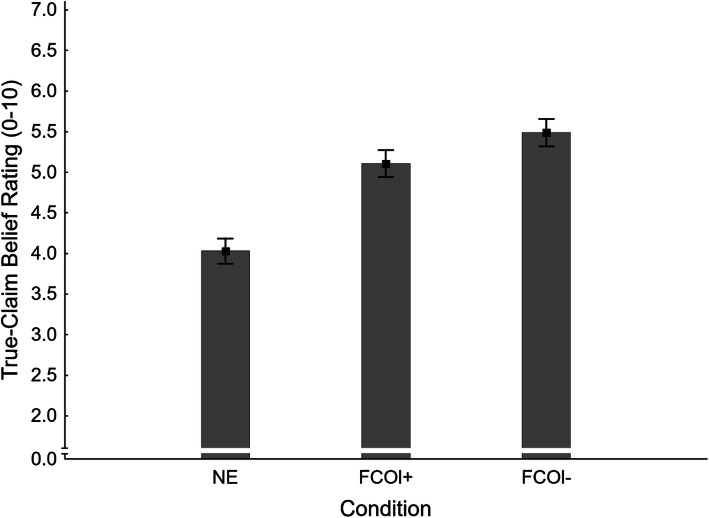
Mean true-claim belief ratings across conditions NE (no-exposure) and FCOl± (fact-check-only, with/without cognitive load) in Experiment 3. Error bars show standard errors of the mean

Mean true-claim belief ratings across conditions are shown in Fig. 15. A one-way ANOVA yielded a significant main effect of condition, *F*(2,405) = 22.32, *η*_*p*_^2^ = .099, *p* < .001. Planned contrasts tested for specific condition differences; results are reported in the fourth panel of Table 3. A mere affirmation increased true-claim belief equally in both load conditions (NE *M* = 4.03, *SE* = 0.16; FCOl- *M* = 5.49, *SE* = 0.17; FCOl+ *M* = 5.11, *SE* = 0.17); this supports H1_TBRl-_ and H1_TBRl+_; it rejects H7_TBR_.

### Discussion

Experiment 3 again found no evidence for familiarity backfire effects in either inference scores or belief ratings. In fact, the no-load condition of Experiment 3 found evidence that a mere correction of a novel claim significantly *reduced* false-claim-congruent reasoning and false-claim belief in a delayed test. Under cognitive load at encoding, a mere correction was unable to reduce misinformed reasoning and beliefs relative to no-exposure control, but also did no harm. In sum, Experiment 3 found no evidence of familiarity backfire and is thus more in line with the findings of Ecker et al. (2020) than the findings of Skurnik et al. (2007). The fact that cognitive load at fact-check encoding reduced the impact of a correction on false-claim inference scores but did not influence the effects of affirmations can be seen as additional evidence that avoiding false-claim-congruent reasoning relies on recollection of the correction, which would have been impaired by the cognitive load (however, no such effect was observed for false-claim belief ratings). Moreover, mere affirmations were generally found to increase true-claim-congruent reasoning and true-claim belief after a delay irrespective of load at encoding, in line with Ecker et al. (2020) and Experiments 1 and 2.

## Bayesian analyses

To further corroborate the evidence for or against familiarity backfire effects, we employed supplementary Bayesian analyses; these have the advantage that evidence in support of a null hypothesis can be quantified (e.g., see Wagenmakers et al., 2018). Specifically, Bayesian ANOVAs were run on inference scores and belief ratings from the no-exposure and fact-check-only conditions of Experiments 1–3 (separately and conjointly; the analysis on Experiment 3 data and the conjoint analysis were pre-registered before running Experiment 3). These tested whether there was evidence for a model including a condition factor over a null model. Mean inference scores across experiments were *M* = 5.06 (*SE* = .05) for the no-exposure condition and *M* = 5.01 (*SE* = .06) for the fact-check only condition (or *M* = 5.12 (*SE* = .06) when using the load condition of Experiment 3). Mean belief ratings across experiments were *M* = 4.40 (*SE* = .08) for the no-exposure condition and *M* = 4.10 (*SE* = .09) for the fact-check only condition (or *M* = 4.15 (*SE* = .08) when using the load condition of Experiment 3).

The Bayes factors (*BF*_10_) in Table 4 quantify the evidence for or against inclusion of the condition factor. A *BF*_10_ > 1 suggests evidence in favor of including a condition factor (which can be interpreted as a main effect of condition); a *BF*_10_ < 1 suggests evidence in favor of the null model. For example, *BF*_10_ = 10 would suggest that the data are 10 times more likely to have occurred under the alternative hypothesis than the null hypothesis; *BF*_10_ = 0.10 would suggest that the data are 10 times more likely to occur under the null hypothesis. *BF* values between 0.33 and 3 are taken to only provide anecdotal evidence; *BF* values between 0.1 and 0.33, or 3 and 10 constitute moderate/substantial evidence; *BF* values < 0.1 or > 10 provide strong to very strong evidence (Jeffreys, 1961; Wagenmakers, Love, et al., 2018).

**Table 4:** Results from Bayesian analyses across Experiments 1–3

dV	Effect direction	*BF*_10_
Experiment 1		
FIS	NE < FCO (familiarity backfire)	2.801
FBR	NE = FCO (no familiarity backfire)	0.154^a^
Experiment 2		
FISd	NE = FCO (no familiarity backfire)	0.135^a^
FBRd	NE = FCO (no familiarity backfire)	0.363
Experiment 3		
FISl-	NE > FCO (corrective effect)	11.757^b^
FBRl-	NE > FCO (corrective effect)	3.065^a^
FISl+	NE = FCO (no familiarity backfire)	0.135^a^
FBRl+	NE = FCO (no familiarity backfire)	0.774
Experiments 1–3		
FIS(l-)	NE = FCO (no familiarity backfire)	0.104^a^
FBR(l-)	NE > FCO (corrective effect)	1.799
FIS(l+)	NE = FCO (no familiarity backfire)	0.112^a^
FBR(l+)	NE = FCO (no familiarity backfire)	0.760

*Note.* FIS and FBR: false-claim inference scores and belief ratings from the delayed test. As test delay was manipulated in Experiment 2, only the delayed-test variables (FISd and FBRd) were entered into analysis. No-load (FISl-; FBRl-) and load (FISl+; FBRl+) conditions of Experiment 3 were included in separate analysis of Experiment 3, and also in separate conjoint analyses. The condition factor includes only conditions NE (no-exposure) and FCO (fact-check-only). ^a^indicates substantial and ^b^indicates strong evidence for or against the null

As can be seen in Table 4, the evidence for a familiarity backfire effect from the inference scores in Experiment 1 was only anecdotal, while Experiment 2 provided substantial evidence against a familiarity backfire effect, and Experiment 3 yielded strong evidence for a *corrective* effect in the no-load condition (which matched the conditions of Experiments 1 and 2), while providing substantial evidence against familiarity backfire in the load condition. Likewise, the secondary belief measures suggested substantial evidence against backfire in Experiment 1 and substantial evidence for a corrective effect in the no-load condition of Experiment 3. However, the main conclusion to be drawn, from the conjoint analyses, is that the experiments reported in this paper overall yielded substantial to strong evidence against familiarity backfire effects: across experiments, while the secondary belief-rating data remained inconclusive, the primary inferential reasoning data were found to be approximately nine times more likely to have occurred under the null hypothesis.

## General discussion

The main focus of this paper was to investigate whether mere exposure to a correction could familiarize people with a novel piece of misinformation such that it would negatively affect their reasoning and beliefs. In other words, we tested whether corrections of novel misinformation could elicit a familiarity-driven backfire effect, which may ironically strengthen misconceptions and spread misinformation to new audiences (Schwarz et al., 2007, 2016).^3^ Experiment 1 found some evidence for a familiarity backfire effect, but the evidence was statistically weak and the result failed to occur in an exact replication with greater experimental power (Experiment 2) as well as a close replication that added only a trivial secondary task (the no-load condition of Experiment 3). In fact, both Experiments 2 and 3 yielded substantial evidence *against* the presence of a familiarity backfire effect, even under conditions that should maximize reliance on familiarity and thus facilitate occurrence of familiarity backfire, viz. the combination of novel claims that maximized the familiarity boost conveyed by first exposure, a relatively long 1-week retention interval, and correction encoding under cognitive load (the load condition of Experiment 3). Thus, while there was some variability across experiments, the overall evidence was in support of the null hypothesis. This meshes well with previous studies failing to find evidence for familiarity backfire with more familiar claims (Ecker et al., 2017, 2020; Swire et al., 2017).

However, this does not rule out misinformation familiarity as an important driver of continued influence effects. This is because we also found consistent evidence that after a delay of 1 week, affirmations of true claims were more effective than corrections of false claims. This closely mirrors the pattern observed by Swire et al. (2017)^4^ and thus corroborates their conclusion that misinformation familiarity can be a counterproductive force when correcting false claims. That is, the overall evidence observed here suggests, in line with Swire et al., that acceptance of false claims can be driven by claim familiarity, in particular when the ability to recollect the correction is reduced (e.g., due to delay-related forgetting or cognitive load). This can offset the correction entirely, such that endorsement of a false claim and false-claim-congruent reasoning can return to baseline after a 1-week delay, which essentially means that even a correction that is reasonably effective in the short term can lose its impact within a week, relative to a no-exposure control condition (as demonstrated in Experiment 2; see Figs. 7 and 8; note that corrections were still somewhat effective relative to the claim-only condition). However, the boost to claim familiarity through claim repetition within the correction is typically not substantial enough to cause actual backfire. Broadly speaking, these results support the view that memory-based evaluation processes determine inferential reasoning and endorsement of claims much more than metacognitive judgments of fluency (cf. Schwarz et al., 2007). The conflicting results from Experiment 1 can only serve as a reminder that one should never place too much emphasis on the findings of a single experiment (e.g., see Murayama, Pekrun, & Fiedler, 2014), and that significant *p* values can translate to only “anecdotal” evidence under a Bayesian framework (see Wagenmakers et al., 2018, for a detailed discussion). We speculate that some of the variability in findings arose due to the use of novel claims. While it was necessary for the present project to use novel claims for the theoretical and practical reasons outlined earlier, the claims we used are not generally representative of claims encountered in the real world, which are typically grounded in contextual world knowledge. Ratings of such novel claims may be inherently less reliable than ratings of familiar claims that can tap into pre-existing knowledge and beliefs (Swire-Thompson, DeGutis, & Lazer, 2020).

Additional evidence obtained in the present set of experiments regards the illusory truth effects conveyed by mere exposure (Begg et al., 1992; Dechêne et al., 2010; Parks & Toth, 2006; Unkelbach, 2007; Weaver et al., 2007). While some research has found that even a single exposure to a false claim can have measurable impact on claim endorsement (e.g., Pennycook et al., 2018), the evidence here was somewhat mixed. Experiment 1 found some evidence for illusory truth effects with true but not false claims, whereas Experiment 2 found evidence for illusory truth effects after a delay with false claims (and also on true-claim belief ratings but not inference scores). This pattern was observed despite the fact that participants could not reliably differentiate between true and false claims, and control-group (no-exposure) belief ratings were generally lower for true claims in both experiments. The fact that illusory truth effects were only observed in the delayed test of Experiment 2 but not in the immediate test suggests that these effects were indeed driven by familiarity rather than perceived social consensus (see Pennycook et al., 2018; Unkelbach, 2007; Weaver et al., 2007). However, apart from that, we can only conclude from these results that a single exposure to a claim can lead to enhanced subsequent endorsement, but that this is not always the case. Thus, to some extent, this mirrors our conclusions regarding the role of familiarity for continued influence, in that the evidence regarding the illusory truth effects that we obtained is somewhat inconsistent, but generally suggests that familiarity likely impacts reasoning and endorsement of claims (we also note that evidence for illusory truth effects in general is much more solid than the evidence for familiarity backfire effects; e.g., see De keersmaecker et al., 2020).

The practical implications of this research are clear: recommendations to front-line educators and communicators to entirely avoid repeating misinformation when debunking (Cook & Lewandowsky, 2011; Lewandowsky et al., 2012; Peter & Koch, 2016; Schwarz et al., 2007, 2016) were unwarranted. Recent research indicates that repeating misinformation when correcting it can have a positive effect, enhancing a correction in the short term (presumably by increasing the salience of the correction and facilitating conflict resolution and knowledge revision processes; see Ecker et al., 2017; Kendeou et al., 2014). There is also evidence that exposure to a correction that repeats a piece of (non-novel) misinformation does not lead to backfire effects relative to either a pre-correction or no-exposure baseline (Ecker et al., 2020). Finally, the present study suggests that exposure to a correction does not cause familiarity backfire relative to a no-exposure control even with novel claims, and thus corrections do not seem to spread misinformation to new audiences easily.

That being said, recommendations to avoid *unnecessary* misinformation repetition should arguably remain in place—while one repetition in the context of a correction may have benefits for correction salience, additional repetition of the misinformation runs the risk of enhancing familiarity without any added benefit. Moreover, while we have demonstrated that corrections do not backfire when it comes to specific beliefs about a proposition, one needs to differentiate this from the over-arching framing that is achieved by stating something that is false (see Lakoff, 2010). For example, a government official stating that there are “no plans for a carbon tax” may achieve a reduction in the specific belief that a carbon tax rollout is being prepared, but at the same time using the word “tax” may make people who oppose new taxes for ideological or pragmatic reasons think about climate change as a threat rather than an opportunity (also see Fletcher, 2009; Kahan, 2010; Lewandowsky et al., 2017). Therefore, communicators should perhaps focus their considerations more on the framing of their corrections, as repeating the misinformation *frame* might do more damage than repetition of the misinformation itself. Investigating the effects of frame repetition within corrections is, therefore, an important target for future research.

## Data Availability

Materials are provided in the Appendix. Data are available on the Open Science Framework website at https://osf.io/69bq3/.

## Appendix

### Claim pilot rating

A total of 100 MTurk participants partook in the online pilot survey; participants could not participate in the main study. Data from participants were excluded for the following two a-priori reasons: (1) uniform responding and (2) completing the survey in less than 5 min. Two participants were classified as uniform responders; across all responses, they showed *SD* < 0.467, the lower outlier criterion of the inter-quartile rule with a 2.2 multiplier. Eight participants completed the survey in less than 5 min. One participant met both criteria. Consequently, *n* = 9 participants were excluded, resulting in a final sample size of *N* = 91 (age range 20–64 years; *M*_age_ *=* 36.48; *SD*_age_ = 10.30; 50 male, 40 female, and 1 participant of undisclosed gender). Claim ratings from the pilot study are presented in Tables 5 and 6 in Appendix.

**Table 5:** Familiarity and believability ratings of false claims in pilot study

Claim	Familiarity		Believability	
	*M*	*SD*	*M*	*SD*
^a^Facebook is about to launch a “no swearing” campaign	1.52	0.95	2.69	1.07
Frequently wearing silk garments in direct contact with the skin can cause spontaneous lactation	1.53	0.96	3.65	0.98
^a^50,000 men were raped in South Africa last year	1.56	1.04	3.66	0.92
NASA is predicting six consecutive days of darkness in the Northern hemisphere in 2022 due to a rare astronomical event	1.57	1.07	3.25	1.06
^a^The ratio of male:female CEOs in Manchester, UK is 1:1	1.60	0.94	3.59	1.03
^a^The outer skin of a pineapple emits a dangerous toxin into the environment when it breaks down	1.60	1.02	2.19	1.19
The Cinderella Castle at Disneyland Florida can be disassembled during hurricanes	1.64	1.04	3.24	1.11
Fibers found in cow skin are now being added to Botox injections	1.76	1.09	2.85	1.00
“Camo” the German shepherd is the only dog in history to become an Officer of the British Empire	1.76	1.07	2.52	1.06
^a^Hugh Hefner donated a fifth of his will to the Planned Parenthood charity	1.84	1.08	2.47	0.99
^a^Placing a car battery on a cement floor can drain it and lead to its decay	1.90	1.28	2.74	1.39
The first artificial intelligence robot has been appointed as a teaching assistant in Japan	2.00	1.11	2.21	0.97
Nike footwear has to meet a quota of containing at least 20% recycled materials	2.10	1.17	3.41	1.32
The motor-vehicle accident rate regularly surges after the Super Bowl in the home state of the losing team	2.15	1.26	3.15	1.06
Bitcoin is used by the American Government as a way to keep track of online criminal activity	2.18	1.41	3.03	0.99
Wireless signals have a direct negative impact on plant growth	2.22	1.28	2.15	1.20
The “redhead gene” is becoming extinct	2.42	1.45	3.60	1.02
Drinking cold water can be bad for your health	2.43	1.48	3.81	1.32
Antibacterial mouthwash helps cure colds and sore throats	2.47	1.41	2.75	1.21
An at-home administration kit to screen for type-1 diabetes is currently being introduced	2.48	1.33	2.52	1.09
Hospitals are busier on full moons	3.10	1.62	2.93	1.36
St Bernard dogs once carried brandy barrels around their necks while rescuing people lost in the mountains	3.43	1.63	2.29	1.01
If you pluck a gray hair, more gray hairs will arrive in its place	3.53	1.54	2.27	0.91
Turkey meat makes you sleepy	3.97	1.58	3.22	0.98

*Note*. ^a^indicates claims used in Experiments 1–3

**Table 6:** Familiarity and believability ratings of true claims in pilot study

Claim	Familiarity		Believability	
	*M*	*SD*	*M*	*SD*
^a^A man in Canada was still allowed to board his flight after a pipe bomb was found in his bag	1.40	0.87	2.29	1.09
In Turkey, people do not chew gum at night due to a superstition that it represents chewing the flesh of the dead	1.40	0.87	2.62	1.03
^a^The color of a chicken’s egg is related to the chicken’s earlobe color	1.51	0.97	2.59	1.03
Hippopotamus milk is pink (item excluded as actually found to be false)	1.54	1.08	2.33	0.87
^a^Chicken carcasses can be used for renewable energy	1.57	1.12	3.38	1.01
2014 was the deadliest year for flying on a plane, with 992 fatalities globally	1.62	0.96	3.27	1.10
^a^The national animal of Scotland is the unicorn	1.68	1.23	2.78	1.08
^a^Saudi Arabia has revealed plans for a US$500 billion “no fossil fuels” mega-city	1.69	1.08	1.98	0.94
Exposure to microwaves can open the blood-brain barrier	1.81	1.10	2.84	1.23
In 2015, Sweden imported nearly 1.3 million tons of waste from Norway, the UK, Ireland, and others	1.84	1.19	2.47	1.28
^a^Pessimism may be inherited due to a genetic mutation	1.93	1.17	2.20	1.01
Dandelion root extract is being tested as a cancer treatment	2.13	1.31	3.43	1.03
After the release of “The Hunger Games” in 2012, women’s participation in archery rose by 105%	2.20	1.37	2.74	1.03
Germany has officially removed any tuition fees for both local and international college students	2.25	1.34	3.57	0.90
Honeybee stings are used in the treatment of arthritis	2.29	1.49	2.75	1.22
The plague is still active in the US today	2.30	1.49	3.00	0.95
26 civilians died in the conflict along the Ukraine-Russia border in the summer of 2017 alone	2.35	1.31	2.78	1.05
The heart of a blue whale is so massive that a human being can swim through its arteries	2.49	1.58	2.97	1.22
Coca Cola single-bottle production is over 110 billion per year	2.52	1.34	3.63	1.37
A mattress doubles its weight after 10 years of usage	2.57	1.56	3.52	1.21
Roughly 800 journalists have been killed globally over the last 10 years	2.59	1.51	2.22	1.23
Nano-robots are being tested in the treatment of cancer	2.78	1.45	3.20	1.32
Acne is a hereditary condition	2.79	1.31	2.75	1.17
China is implementing a citizen ranking system to determine who is a good citizen	3.47	1.71	2.21	1.29

*Note*. ^a^indicates claims used in Experiments 1–3

### Inferential-reasoning questions (R = reverse-coded)

#### False claims

1. Facebook does not care about the language used on its platform (R)
2. Facebook is investing money into promoting inoffensive language on its platform
3. Men in South Africa generally do not need to be concerned about sexual assault (R)
4. The rising number of reported HIV cases in South Africa is partially due to a large number of male rape cases
5. Industries concerned about gender equity can look to Manchester, UK for solutions
6. In Manchester’s corporate environment, males are much more likely than females to be promoted to senior managerial positions (R)
7. There should be an awareness campaign to educate consumers about the environmental risks associated with pineapple skin
8. There is no need to worry about how to dispose of pineapple skin (R)
9. Hugh Hefner was bankrupt when he died (R)
10. The late Hugh Hefner was a philanthropist, supporting various charities
11. Concrete has no impact on electronics (R)
12. When taking out the battery of your car, it is important not to place it on a concrete floor

#### True claims

1. Canada is lax regarding security on commercial flights
2. In Canada, suspected terrorists are given an immediate ban on flying (R)
3. A farmer can look at a chicken and predict the color of the egg (white or brown) it will lay
4. Whether a chicken’s egg is white or brown is completely random (R)
5. The only profitable use of chickens lies in meat and egg production (R)
6. In the future, it is likely that some of our energy will come from bio-matter such as animal remains
7. Souvenir shops in Scotland are likely to stock unicorn figures
8. Scotland’s national animal can be found in most zoos (R)
9. Saudi Arabia is investing billions of dollars into environmental sustainability
10. Investment in renewable energy technology in Saudi Arabia is virtually non-existent (R)
11. In the future, genetic testing will be able to tell you if your baby will grow into a pessimistic person
12. Whether people become pessimistic depends entirely on their life experiences (R)

## Footnotes

1 Technically, the design was a 2 × 2 × 2 mixed design with the within-subjects factor claim veracity (true/false); however, as the prime interest was on false claims, analyses were conducted separately for true and false claims.

2 Subscripts FIS, FBR, TIS, TBR will be used to refer to false-claim inference scores, false-claim belief ratings, true-claim inference scores, and true-claim belief ratings, respectively.

3 We note that Kessler, Braasch, and Kardash (2019) recently reported a backfire effect with vaccination misinformation, which they observed only in people with high “flexible thinking” scores. Kessler et al. speculated that in flexible thinkers—those who open-mindedly consider new information—corrections might thus spread novel misinformation. However, they did not measure misinformation novelty, and only prior vaccination *beliefs* and not prior vaccination *knowledge* predicted the backfire effects that they observed; it therefore seems more likely that these effects were driven by worldview rather than familiarity (see Ecker & Ang, 2019; Lewandowsky et al., 2012).

4 Peter and Koch (2016) also observed this asymmetry, although they referred to this asymmetry itself as a familiarity backfire effect, which, in our view, is a misnomer.
